# Determinants of heritable gene silencing for KRAB-dCas9 + DNMT3 and Ezh2-dCas9 + DNMT3 hit-and-run epigenome editing

**DOI:** 10.1093/nar/gkac123

**Published:** 2022-03-02

**Authors:** Henriette O’Geen, Marketa Tomkova, Jacquelyn A Combs, Emma K Tilley, David J Segal

**Affiliations:** Genome Center, University of California, Davis, CA 95616, USA; Genome Center, University of California, Davis, CA 95616, USA; Genome Center, University of California, Davis, CA 95616, USA; Genome Center, University of California, Davis, CA 95616, USA; Genome Center, University of California, Davis, CA 95616, USA; Department of Biochemistry and Molecular Medicine, University of California, Davis, CA 95616, USA

## Abstract

Precision epigenome editing has gained significant attention as a method to modulate gene expression without altering genetic information. However, a major limiting factor has been that the gene expression changes are often transient, unlike the life-long epigenetic changes that occur frequently in nature. Here, we systematically interrogate the ability of CRISPR/dCas9-based epigenome editors (Epi-dCas9) to engineer persistent epigenetic silencing. We elucidated *cis* regulatory features that contribute to the differential stability of epigenetic reprogramming, such as the active transcription histone marks H3K36me3 and H3K27ac strongly correlating with resistance to short-term repression and resistance to long-term silencing, respectively. H3K27ac inversely correlates with increased DNA methylation. Interestingly, the dependance on H3K27ac was only observed when a combination of KRAB-dCas9 and targetable DNA methyltransferases (DNMT3A-dCas9 + DNMT3L) was used, but not when KRAB was replaced with the targetable H3K27 histone methyltransferase Ezh2. In addition, programmable Ezh2/DNMT3A + L treatment demonstrated enhanced engineering of localized DNA methylation and was not sensitive to a divergent chromatin state. Our results highlight the importance of local chromatin features for heritability of programmable silencing and the differential response to KRAB- and Ezh2-based epigenetic editing platforms. The information gained in this study provides fundamental insights into understanding contextual cues to more predictably engineer persistent silencing.

## INTRODUCTION

All cells within a multicellular organism have almost identical DNA content, but the transcriptional program is spatiotemporally regulated. It is now appreciated that epigenetics plays a fundamental role in the regulation of gene expression of genomic DNA content during normal and pathological development. Epigenetic factors include chromatin accessibility, DNA modifications, post-translational modifications of histones and 3D genome organization (1–3). Distinct epigenetic landscapes define genomic function and cellular identity. For example, tri-methylation of lysine 4 on histone 3 (H3K4me3) and H3K27 acetylation (H3K27ac) decorate active promoters, while promoters of repressed genes are typically associated with the repressive marks H3K27me3 or DNA methylation of cytosine 5 (5-mC) (4–6). Epigenetic marks are stably inherited from one generation to the next but are reversible upon developmental cues or external stimuli. Bivalent genes are a unique set of genes poised for transcription that has been identified by the presence of active (H3K4me3) and repressive (H3K27me3) histone marks at the same promoter (7,8). Upon developmental cues bivalent genes transition from a silenced to an activated epigenetic state that allows dynamic regulation of gene expression during development and cell differentiation. On the other hand, environmental exposures (prenatal stress, traumatic events and exposures to certain toxins) can alter epigenetic information, leading to persistent pathologic changes in gene expression (9). Dysregulation of epigenetics can result in persistent pathological states of gene expression including heart disease, neurologic disorders, cancer development and progression (10–14).

Both the ability to adapt to developmental and external cues and the heritability of epigenetic states are important aspects of epigenetics. It is the reversibility of epigenetic features that enables intervention in epigenetic disease through epigenome editing. With the RNA-guided Cas9/CRISPR complex, we now have a tool that can easily and precisely target a 20-bp sequence in the genome (15–17). Targeted epigenetic regulators (Epi-dCas9) are based on fusions of epigenetic effector domains to a catalytically inactive dCas9 platform and can alter epigenetic marks in a targeted manner without altering genetic information (18–22). Longevity and heritability of induced epigenetic changes and associated expression changes have not been comprehensively studied. We and others have shown that targeting dCas9 fused to a single effector domain is rarely sufficient to induce a long-term repressed state, but a combination of dCas9-fusions to transcriptional repressors (KRAB-dCas9) and DNA methyltransferases (DNMT3A-dCas9 + DNMT3L) is required for engineering robust and stable silencing (23–26). Recently, the CRISPR silencing platform has been improved by creating a single dCas9 fusion with the same effector domains (KRAB and DNMT3A-3L) and demonstrated heritable expression changes at a GFP reporter (27) and at many genes genome-wide (28), although the authors pointed out that it remains unknown how many genes are amenable to stable silencing. There is a need to identify *cis* regulatory features that dictate the heritability of programmed epigenetic memory. Our study aimed to address exactly this limitation.

Mammalian cells have two different epigenetic mechanisms for repressed chromatin, also referred to as heterochromatin. Constitutive heterochromatin is stably inherited and is associated with H3K9me3 and DNA methylation, while facultative heterochromatin is reversible and depends on developmental stage or cell type. The KRAB repressor domain recruits KAP1/SETDB1 co-repressor and the NuRD nucleosome remodeling and deacetylase complex, while the H3K27 histone methyltransferase EZH2 recruits the PRC2 complex for deposition and PRC1 complex for maintenance of H3K27me3. KRAB and Ezh2 offer complementary capacity in engineering long-term silencing via constitutive and facultative heterochromatin formation, respectively. We previously demonstrated that targeting DNA methyltransferases (DNMT3A-dCas9 + DNMT3L) and either (KRAB-dCas9) or (Ezh2-dCas9) lead to transient silencing at the *HER2* promoter, but only Ezh2-dCas9 facilitated persistent *HER2* silencing while KRAB allowed re-activation (25). Clearly, a better understanding is needed of the targetable epigenome that is amenable to persistent gene silencing and an understanding of the pathway(s) to accommodate persistent gene silencing.

Here, we systematically interrogated the *cis* regulatory features that affect the ability of a combination of CRISPR-based epigenome editors to engineer a persistent epigenetic change at 24 endogenous loci. We show that fusions of dCas9 to transcriptional repressors (KRAB-dCas9 or Ezh2-dCas9) and DNA methyltransferases (DNMT3A-dCas9 + DNMT3L) were very efficient in transient repression of target genes, but persistent hit-and-run silencing occurred at a subset of target genes. We integrated genome-wide chromatin data to elucidate the role of chromatin microenvironment on the differential amenability of genes to heritable epigenetic reprogramming. In particular, we identified a strong correlation between the histone modification H3K27ac and resistance to persistent silencing by KRAB/D3A + L epigenetic editing (KRAB-dCas9 and DNA methyltransferases DNMT3A-dCas9 + DNMT3L). In addition to H3K27ac, enrichment of RNA polymerase (POLR2A) and insulator CTCF at the target region predicted resistance to permanent transition from an active/open chromatin state to a silenced/closed chromatin state. The increase in engineered DNA methylation inversely correlates with pre-existing H3K27ac levels at the target locus. Intriguingly, gene promoters that displayed divergent chromatin states in other cell types were more accessible to persistent silencing by KRAB/D3A + L. These same dependencies were not observed when using the Ezh2/D3A + L epigenetic editing platform, supporting the conclusion that alternative epigenetic editor combinations may need to be used depending on local chromatin environments. Notably, co-targeting of Ezh2 and DNA methyltransferases had an enhanced effect on engineering localized DNA methylation that provides us with the perfect set of tools to further our mechanistic understanding of their crosstalk.

In summary, our analysis of 24 promoters identified distinct sets of chromatin features associated with the ability to engineer short-term and long-term silencing. Insight gained from our findings will guide future epigenetic engineering experiments and help develop conceptual models towards understanding and predicting outcomes of targeted epigenetic engineering.

## MATERIALS AND METHODS

### Plasmid construction

Plasmids expressing dCas9 fusions with KRAB, Ezh2, DNMT3A effector domains, as well as the dCas9 cloning vector without any effector domain, have previously been described (25,29) and are available through Addgene (KRAB-dCas9 #112195, Ezh2-dCas9 #100086, DNMT3A-dCas9 #100090). The DNMT3L expression plasmid pCDNA-DNMT3L was a kind gift from Dr Fred Chedin (30). DNMT3A-dCas9 (Addgene #100090) and pCDNA-DNMT3L are used in combination and are abbreviated to D3A + L in this study. CRISPRoff v2.1 was a gift from Luke Gilbert (Addgene plasmid # 167981) (28). gRNA_Cloning Vector was a gift from George Church (Addgene plasmid # 41824) (31). Four 19-bp gRNA target sequences were selected to be as evenly spaced as possible within 250 bp of each relevant gene promoter using the online tool CHOPCHOP v2 (32). STAT5A was an exception and was targeted with only 3 gRNAs. Each gRNA sequence was cloned as G-N19 into the *Afl*II-linearized plasmid using Gibson cloning. The gRNA sequences used to create target-specific vectors are listed in Supplementary Table S1.

### Cell culture and transfection

K562 (ATCC #CCL-243) cells were maintained in RPMI-1640 supplemented with 10% FBS and 1× Penicillin/Streptomycin at 37 °C under 5% CO_2_. A total of 2 × 10^6^ cells were transiently transfected using the Neon Transfection System (ThermoFisher) following the manufacturer’s instructions (Neon settings: 1450 V, 10 ms, 3 pulses). About 5 μg of combinations of dCas9 expression vectors were co-transfected with 5 μg of equimolar pooled gRNA expression vectors using 100 μl tips. Transfected cells were plated and maintained in 6-well plates in 3 ml media with routine transfection efficiencies >95% (Supplementary Figure S1A). We did not observe significant amounts of toxicity related to transfection. Cells were split every 3–4 days at an approximate density of 2 × 10^5^ cells. HeLA S3 (ATCC # CCL-2.2) cells were maintained in Dulbecco’s modified Eagle’s medium (DMEM) supplemented with 10% FBS and 1× Penicillin/Streptomycin at 37°C under 5% CO_2_. Cells of 60–70% confluency were transfected using Lipofectamine 3000 (Life Technologies) following the manufacturer’s instructions. Briefly, transfections were performed in 12-well plates using 625 ng containing combinations of epi-dCas9 and DNMT3L expression vectors and 500 ng of equimolar pooled gRNA expression vectors. Cells were co-transfected with 125 ng of puromycin-resistant plasmid pBABE-puro to select for transfected cells. Transfection medium was replaced 48 h post-transfection with growth medium containing 3 μg /ml puromycin to enrich for transfected cells. After 72 h of puromycin selection, media were switched to standard growth media. Cells from three biological replicates were pelleted 4 and 21 days after transfection for short- and long-term timepoints, respectively. RNA and genomic DNA were extracted from freshly pelleted cells or cell pellets previously frozen at −80°C using the Quick-DNA/RNA Miniprep Plus kit (Zymo Research).

### Reverse-transcription quantitative PCR (RT-qPCR)

RNA was reverse transcribed using the AB high-capacity cDNA synthesis kit (ThermoFisher) according to the manufacturer’s instructions. Primers for quantitative PCR (qPCR) were designed using Primer3 (33). RT-qPCR was performed in triplicate using PowerUp SYBR Green Master Mix (ThermoFisher) with the CFX384 Real-Time System C1000 Touch system (Bio-Rad). Gene expression analysis was performed with gene specific primers (Supplementary Table S1) using three biological replicates. Relative target gene expression was calculated as the difference between the target gene and the GAPDH reference gene (dCq = Cq[target] − Cq[GAPDH]). Gene expression results are indicated as fold change to a reference sample (dCas9 combination without targeting gRNA), using the ddCq method. Multiple unpaired *t*-tests were used to determine statistical significance for individual loci and *P*-values were adjusted using Benjamini–Hochberg (FDR 0.05) correction (*q-*values).

### Chromatin immunoprecipitation (ChIP) and ChIP-qPCR

K562 cells were transfected with plasmids expressing D3A-dCas9 and D3L and either KRAB-dCas9 or Ezh2-dCas9 and four gRNAs targeting individual promoters. Control cells were transfected with mCherry. Cells were cross-linked for 10 min in 1% formaldehyde 4 days after transfection for short-term repression timepoints and 21 days after transfection for long-term silencing timepoints. Cross-linking was stopped with 0.125 M glycine, washed in DPBS and cell pellets were stored at −80°C. Cross-linked cells were lysed with ChIP lysis buffer (5 mM PIPES pH 8, 85 mM KCl, 1% Igepal) with a protease inhibitor (PI) cocktail (Roche). After centrifugation at 2000 rpm for 5 min at 4°C nuclear pellets were lysed in nuclei lysis buffer (50 mM Tris pH 8, 10 mM EDTA, 1% SDS) supplemented with PI cocktail. Chromatin was fragmented using the Bioruptor Pico (Diagenode) with 4 cycles of 30 s on and 30 s off and diluted with 5 vol RIPA buffer (50 mM Tris pH 7.6, 150 mM NaCl, 1 mM EDTA pH 8, 1% Igepal, 0.25% deoxycholic acid). About 10% of chromatin was retained as input control. ChIP assays were carried out for 16 h at 4°C with 2 μg H3K9me3 antibody (Diagenode C15410056), 2 μg H3K27me3 antibody (Diagenode C15410195) or 2 μg H3K27ac antibody (Active Motif #39133). Immune complexes were captured with 20 μl magnetic protein A/G beads (ThermoFisher) for 2 h at 4°C. Beads were washed 2× with RIPA, 3× with ChIP wash buffer (100 mM Tris pH 8, 500 mM LiCl, 1% deoxycholic acid) and once with ChIP wash buffer plus 150 mM NaCl. ChIP complexes were eluted in 100 μl ChIP elution buffer (50 mM NaHCO_3_, 1% SDS) for 30 min and cross-links were reversed in presence of 0.5M NaCl overnight at 65°C. ChIP DNA was purified using the QIAquick PCR Purification Kit (Qiagen). Quantitative PCR was performed on ChIP and input control samples with 2 × SYBR FAST mastermix (KAPA Biosystems) using the CFX384 Real-Time System C1000 Touch Thermo Cycler (BioRad). ChIP amplification primers are listed in Supplementary Table S1. ChIP enrichment was calculated relative to input samples using the dCq method (dCq = Cq[ChIP] − Cq[input]). To quantitatively determine reduction of H3K27ac at target sites, ddCq was determined by normalization to the GAPDH promoter (ddCq = dCq[target]/dCq[GAPDH]). Statistical significance was determined using Dunnett’s multiple comparisons test.

### Targeted DNA methylation analysis

All experiments were performed from three independent biological replicates. About 250–500 ng genomic DNA was bisulfite converted using the EZ DNA Methylation-Lightning kit (Zymo Research). Bisulfite-Sequencing PCR (BSP) amplification of 25–50 ng of bisulfite converted ssDNA was carried out with ZymoTaq DNA polymerase (Zymo Research) according to manufacturer’s instructions. BSP primers were designed using MethPrimer 2.0 (http://www.urogene.org/methprimer2/;^34^) to amplify an average target region between 200 and 250 bp. Setting for degenerate primers was used when MethPrimer 2.0 was unable to identify primers around the target region. BSP primers are listed in Supplementary Table S1. Forward primers contain a unique 6-nucleotide barcode at the 5’ end of the sequence. BSP amplicons were purified using the QIAquick PCR purification kit (QIAGEN). Equal amounts of purified BSP amplicons were pooled. Library preparation and PE150 sequencing (CRISPR sequencing) were performed by the CCIB DNA Core Facility at Massachusetts General Hospital (Cambridge, MA). After demultiplexing of sequence read files, forward and reverse reads were merged into a single long read using FLASH2 (35). Processed fastq files were aligned and cytosine methylation states determined using Bismark (36). Only samples with at least 100× coverage were used for downstream analysis. In the visualization on genome, the raw beta-values (percentage of unconverted reads) are shown, as well as smoothed signal (MATLAB function polyfit with the degree of 15). For read-level DNA methylation analysis, the percentage of methylated positions was computed for each read. The first 19 bp and the last 1 CpG were trimmed to prevent technical methylation bias and data from all replicates were pooled for this analysis. A distribution curve was fitted using MATLAB function ksdensity (parameters: pts 0:0.1:100 and bandwidth 5). The number of modes of the distribution was then identified using MATLAB function findpeaks (parameters: MinPeakProminence 0.002). The *STAT5A* region was excluded due to having only 3 CpG positions in the read.

### Chromatin correlation analysis

Previously published epigenome data from K562 was obtained from ENCODE and UCSC (Supplementary Table S2). All data sets were in hg19 or converted to hg19 using liftover tool by UCSC. Average values in the target regions (250 bp) and the extended regions (250 bp ± 1 kb) were computed using bedtools map. The baseline genomic and epigenomic features were compared in their ability to predict the extent of repression of the corresponding genes: the expression at 4 and 21 d, respectively, relative to the baseline (i.e., expression at 4 d/expression at baseline; expression at 21 d/expression at baseline), as measured by RT-qPCR. Spearman correlation between the features and the relative expression was used to assess the predictivity, and Benjamini–Hochberg method (matlab function mafdr) was used to correct for multiple testing. The resulting *q*-values <0.05 were considered significant (***q < 0.001; **q < 0.01; *q < 0.05).

### Chromatin states analysis

Chromatin states values of chromHMM model (37) for 9 ENCODE cell lines (K562, GM12878, H1 ES, HepG2, HMEC, HSMM, HUVEC, NHEK, NHLF) were downloaded from UCSC (Supplementary Table S2). For each target region of the 24 genes and each of the 9 cell lines, we computed which of the 15 states in the chromHMM model are present in the region using bedtools. In some regions, more than one state was present. Subsequently, we evaluated whether presence of the individual chromatin states across cell lines can predict the extent of repression in K562 cell line. In particular, for each of the 15 states, we correlated the percentage of cell lines with this state present in the target region and the relative gene expression at 21 days compared to baseline using Spearman correlation followed by Benjamini–Hochberg multiple testing correction.

### Availability of data and materials

All plasmids used in this study are available on Addgene. Genome-wide datasets used in this study are listed in Supplementary Table S2. All data that led to the conclusions of this study are available in Supplementary Figures and Tables.

## RESULTS

### Baseline gene expression levels predict short-term but not long-term silencing by KRAB/D3A + L epi-dCas9

**Figure 1.**
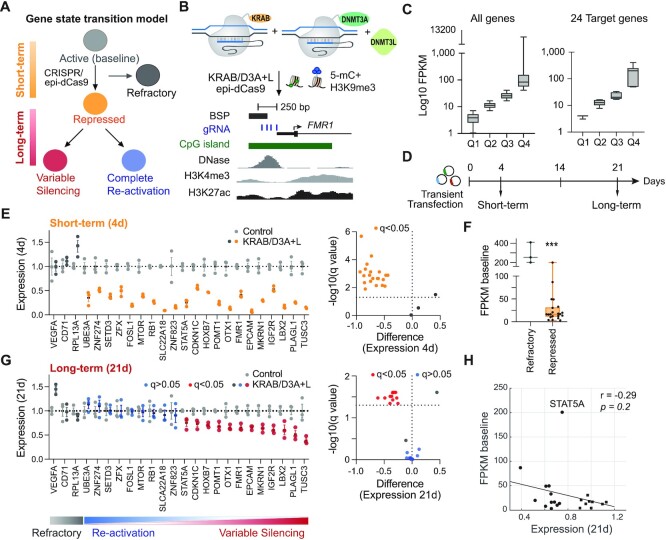
Gene-specific hit-and-run epigenetic editing by KRAB/D3A + L epi-dCas9. (**A**) Model of gene state transition of target gene to short- or long-term silencing in response to transient epigenetic editing. Refractory genes escape repression. (**B**) Diagram depicting epigenetic editing at a representative active promoter (*FMR1*). Epi-dCas9/gRNA complex acts as DNA binding module and is fused to epigenetic effector domains (ED) responsible for deposition of epigenetic marks. 4 guide RNAs (blue bars) target promoter -1 to -250 nucleotides upstream of the TSS. UCSC genome browser tracks for K562 cells are shown for CpG islands (green), DNase hypersensitivity, H3K4me3 and H3K27ac. (**C**) Distribution of all expressed K562 genes from ENCODE RNA-seq data (FPKM > 1). 24 target genes used in this study represent these 4 quartiles (Supplementary Table S3). (**D**) Experimental design indicates transient transfection of epi-dCas9 and gRNA expressing plasmids. Cells were analyzed 4 days (short-term) and 21 days (long-term) after transfection. (**E**) Short-term repression was evaluated by RT-qPCR (*n* = 3, error bar is ± SEM; multiple unpaired *t*-tests with Benjamini–Hochberg correction) 4 days after transient transfection. Relative gene expression has been normalized to *GAPDH* and is plotted relative to control transfection with non-targeting gRNA. Volcano plot evaluates the difference of *GAPDH* normalized expression between KRAB/D3A + L (KAL) treatment and control at 4 days. Difference between mean values is plotted along *x*-axis. Refractory genes are dark gray and short-term repressed genes are orange. The horizontal dotted line indicates *q = 0.05* on the *y*-axis of volcano plot. (**F**) Box plot evaluates differences between refractory and short-term repressed genes (***P< 0.001). (**G**) Long-term (heritable) silencing was determined by RT-qPCR (*n* = 3, error bar is ± SEM; multiple unpaired *t*-tests with Benjamini–Hochberg correction) 21 days after transfection. Variably silenced (red, *P< 0.05) and re-activated genes (blue, *P* > 0.05) are indicated. Volcano plot evaluates the difference of *GAPDH* normalized expression between KRAB/D3A + L (KAL) treatment and control at 21 days. Difference between mean values is plotted along *x*-axis. Genes with long-term silencing are red and re-activated genes are blue. The horizontal dotted line indicates *q*= 0.05 on the *y*-axis of volcano plots. (**H**) Spearman correlation plot determined lack of correlation between baseline expression (FPKM) and long-term silencing.

Transcriptional regulators, such as KRAB-dCas9 fusions, are commonly used for transient repression of specific target genes, but a combination of epigenetic effector domains is often required for persistent silencing. Although combinatorial epi-dCas9 treatment induces the epigenetic change in gene expression, endogenous cellular processes are required to maintain this altered epigenetic state after synthetic regulators are no longer present. We hypothesized that studying a large set of endogenous target genes could identify factors that could potentially distinguish genes that are amenable to engineering persistent silencing and genes that are not (Figure 1A). We used K562 cells as our model cell line, which allowed us to take advantage of publicly available data sets (6). Using available RNA-seq data, we stratified all K562 genes into quartiles based on their expression levels (baseline FPKM) and chose 24 genes distributed across all four expression quartiles (Figure 1C and Supplementary Table S3). Genes with FPKM > 1 were considered expressed. We used a combination of dCas9-fusions (KRAB/D3A + L epi-dCas9) with transcriptional repressors (KRAB-dCas9; K) and DNA methyltransferases (DNMT3A-dCas9 + DNMT3L; D3A + L) to target 24 active promoters in their endogenous cellular context (Figure 1B). The KRAB effector domain recruits the KAP1/SETDB1 co-repressor complex depositing H3K9me3 at the target site, and cooperates with the DNA-methylation machinery to deposit 5-methylcytosine (5-mC) DNA methylation and thereby represses gene expression. Each gene was targeted with four gRNAs to the 250 bp promoter region immediately upstream of the transcription start site (TSS, Figure 1B). Plasmids expressing the KRAB/D3A + L epi-dCas9 combination and the 4 gRNAs (Supplementary Table S1) were co-transfected with routine transfection efficiencies >95% (Supplementary Figure S1A). Expression of each gene was determined by RT-qPCR and compared to control cells without gRNA using multiple unpaired *t*-tests. *P*-values were adjusted (*q*-value) using Benjamini and Hochberg correction. ‘Short-term’ repression was evaluated four days post transfection, while cells were maintained for 21 days before assessing ‘long-term’ or persistent silencing (Figure 1D). We and others (15,25,28) have previously demonstrated that epi-dCas9 regulators are no longer detectable 10 days post transfection (Supplementary Figure S1B). The control dCas9 without epigenetic editing domains was unable to elicit persistent silencing (Supplementary Figure S1D).

As expected, the epi-dCas9 combination of KRAB/D3A + L was efficient in reducing expression 2–14-fold at most target genes (21 out of 24, *q* < 0.01) at the short-term timepoint (after 4 days; Figure 1E, Supplementary Table S3). Three genes (*VEGFA, RPL13A* and *CD71*) were refractory to the treatment. Baseline expression of refractory genes (median FPKM = 255, IQR 215–371) was significantly higher than that of repressed genes (median FPKM = 16, IQR 12–31) (rank-sum test *P =*0.007, Figure 1F). Long-term expression was evaluated 21 days after transfection. We observed two distinct groups. Nine of the 21 short-term repressed genes were ‘re-activated’ to baseline expression levels (*q* > 0.6, Figure 1G). In contrast, none of the remaining 12 genes returned to baseline expression level by 21 days (*q* < 0.05), and instead displayed ‘variable silencing’, in which some genes showed partial return to baseline that generally stabilized by day 14 and others exhibited stable silencing across all timepoints (Supplementary Figure S1C and D). Baseline FPKM was not significantly different between variably silenced genes (median FPKM = 18, IQR 12–49) and re-activated genes (median FPKM = 16, IQR 10–19) (rank-sum test *P* = 0.3). Neither baseline expression (Spearman correlation *P* = 0.2; Figure 1H) nor relative gene expression at 4 days (short-term) (Spearman correlation *P* = 0.2) correlated with long-term silencing.

In conclusion, high baseline gene expression predicts escape from short term repression by epi-dCas9 KRAB/D3A + L, but neither baseline expression nor short-term repression levels can predict whether a gene is amenable to long-term silencing.

### Short-term DNA methylation by KRAB/D3A + L predicts neither long-term DNA methylation nor short-term or long-term silencing

Although KRAB-dCas9 can efficiently repress transcription, targeted DNA methylation (DNMT3A-dCas9 + DNMT3L) is often required for persistent hit-and-run epigenome editing (23,25,29). CpG islands (CGIs) located proximal to transcriptional start sites (TSS) are typically unmethylated and are associated with a transcriptionally permissive state. However, CGI promoters can become silenced through DNA methylation or PRC2-mediated H3K27 tri-methylation (38). Targeted DNA methylation so far has shown variable effects on gene expression and often results in modest or no repression, reviewed in (19). This highlights challenges of epigenome editing and the need for a better understanding how to achieve significant and long-lasting gene expression changes.

**Figure 2.**
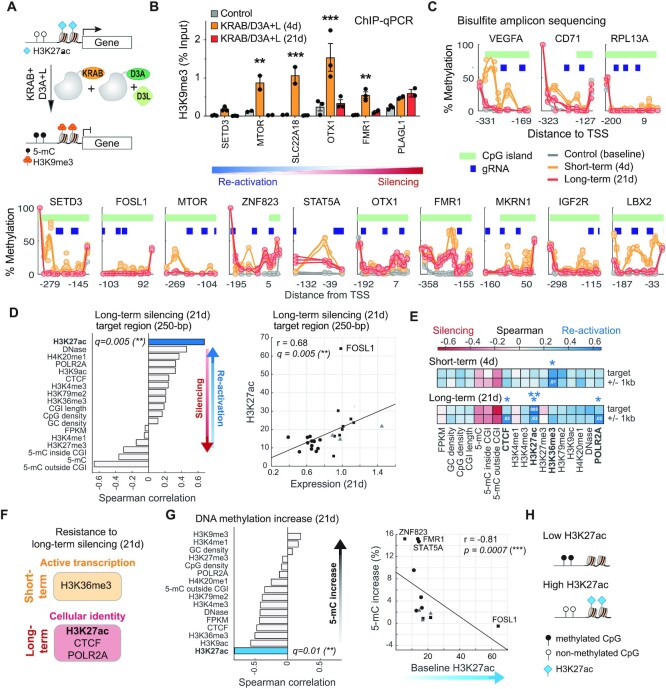
Distinct *cis* chromatin features correlate with short- and long-term silencing by KRAB/D3A + L epi-dCas9. (**A**) Cartoon depicting silencing of target genes by engineering histone and promoter methylation. (**B**) H3K9me3 ChIP-qPCR enrichment was calculated relative to input at 4 and 21 days after transfection with plasmids expressing KRAB/D3A + L epi-dCas9 and 4 gRNAs per target gene. H3K9me3 enrichment was compared to control cells transfected with dCas9 without epigenetic editing domains (*n* = 3, error bar is ± SEM, Dunnett’s multiple comparisons test **P< 0.01, ***P< 0.001). (**C**) Methylation profiles were determined at baseline, 4 and 21 days after transfection using next-gen bisulfite amplicon sequencing (*n* = 3). Each circle represents a single CpG and pale green bars represent CpG islands. Blue boxes depict gRNA target sites. Distance relative to TSS is shown on the *x*-axis. Methylation plots are shown with refractory genes at the top. Genes in the bottom panel are arranged from highest level of re-activation on the left to highest level of persistent silencing on the right (as determined in Figure 1). (**D**) Correlative analysis of long-term silencing with enrichment of chromatin features in the 250-bp target region (**q< 0.01; Spearman correlation with Benjamini–Hochberg correction). Scatterplot of H3K27ac showing significant Spearman correlation (**q< 0.01) with persistent silencing. Positive Spearman correlation r indicates correlation with re-activation of target genes. (**E**) Heat map of Spearman correlation values after Benjamini–Hochberg correction for short- and long-term repression with enrichment of chromatin features in the 250 bp target region and flanking region (250-bp target region ± 1 kb). Significant adjusted *P*-values (*q < 0.05, **q< 0.01) are indicated with asterisk and significant *q*-values are highlighted inside heatmap. (**F**) Summary of distinct chromatin features correlating with resistance to short-term repression and long-term silencing. (**G**) Correlative analysis of engineered DNA methylation (5-mC) increase with enrichment of chromatin features in the 250-bp target region (*q =0.01; Spearman correlation with Benjamini–Hochberg correction). Scatterplot of H3K27ac shows significant inverse correlation (*q= 0.01) with 5-mC increase. (**H**) Diagram summarizing the observed relationship between H3K27ac and engineering 5-mC increase.

To evaluate the impact of engineered histone H3K9 trimethylation and DNA methylation on gene expression (Figure 2A), we performed ChIP-qPCR assays and targeted bisulfite amplicon sequencing on a subset of target genes. We chose three representative genes that were considered re-activated (*SETD3, MTOR, SLC22A18*) and three genes that showed significant levels of long-term silencing (*OTX1, FMR1, PLAGL1*). We confirmed that a H3K9me3 ChIP positive control region (*ZNF554*) was equally enriched in untreated control cells and treated cells (Supplementary Figure S2A). Although four of the six genes (*MTOR, SLC22A18, OTX1, FMR1*) showed strong short-term H3K9me3 enrichment at 4 days, H3K9 tri-methylation returned to baseline levels comparable to untreated control cells by 21 days (Dunnett’s multiple comparisons test; Figure 2B). We have previously observed short-term bursts of H3K9me3 when targeting the *HER2* promoter in HCT116 cells (25). Since engineered H3K9 tri-methylation was not heritable, we hypothesized that the increase of DNA methylation induced by epi-dCas9 (KRAB/D3A + L) and maintained by cellular processes would inversely correlate with gene expression (Figure 2A). We amplified ∼250-bp regions in the proximal promoter of 13 representative target genes and performed targeted bisulfite amplicon sequencing (Figure 2C). DNA methylation was measured at three timepoints using biological triplicates: (i) baseline control methylation at day 0, (ii) engineered *de novo* methylation at day 4 and (iii) persistent methylation at 21 days after transient treatment with KRAB/D3A + L epi-dCas9. DNA methylation was calculated for each target gene as the average methylation of all CpGs within the ∼250-bp amplicon region. The increase in DNA methylation was then established relative to baseline methylation at day 0 (Supplementary Table S3). Baseline methylation of control (d0) ranged from 1 to 12%. Most target promoters (11 of 13) showed >5% increase in DNA methylation 4 days after transfection, but magnitude of increase varied widely (9–25%; Figure 2C, Supplementary Figure S2B). Target regions of *RPL13A* and *FOSL1* showed <5% methylation increase. It is worth noting that an initial DNA methylation increase was observed at the refractory genes *VEGFA* (20.5%) and *CD71* (8.6%), but did not result in reduced gene expression. After the initial burst of DNA methylation (4d), DNA methylation decreased and by 21 days only 4 of 13 genes retained >5% methylation above baseline control (Supplementary Figure S2C). Overall, we observed mostly transient DNA-methylation (median DNA methylation = 11.9, IQR 8.2–20.6, Supplementary Figure S2C) that was not stable, reflected in a methylation drop-off by 21 days (Wilcoxon ranksum test *P* = 0.003; median DNA methylation = 2.3, IQR 1.0–12.1, Supplementary Figure S2D). We did not detect any significant correlation between methylation increase and short- or long-term silencing (Spearman correlation *P* = 0.5 and *P* = 0.09, respectively; Supplementary Figure S2E). However, it is worth noting that genes clearly reactivated at 21 days have low 5-mC increase, but genes that are reluctant to reactivation show a wide range of methylation increase, suggesting that persistent methylation increase is required but not sufficient on its own for persistent silencing.

We next leveraged read-level methylation analysis to obtain a more complete picture of DNA methylation across cells and computed the distribution of methylated positions per read (Supplementary Figure S3). All but one gene (*LBX2*) displayed unimodal distribution of short-term methylation (4d). Unimodal distribution indicates stochastic methylation at certain fractions of available CpG positions (Supplementary Figure S3A) rather than distinct populations of unmethylated and methylated events. Highest mode methylation for unimodal distribution was observed for *VEGFA* (26%), *SETD3* (17%) and *FMR1* (21%) compared to controls (0–1%). Although *VEGFA* demonstrated highest mode methylation, the gene was refractory to short-term repression (Figure 1G). In concordance with our previous observations (Supplementary Figure S2D), a general loss of DNA methylation was detected in all regions between 4 and 21 days after transient treatment with KRAB/D3A + L epi-dCas9 (Supplementary Figure S3B). All 12 tested genes showed unimodal distribution of long-term methylation with the bulk of reads being unmethylated and stochastic methylation of the remaining reads. Although combinatorial KRAB/D3A + L epi-dCas9 treatment is very effective at inducing a transient epigenetic change and altering gene expression, it is not predictive of its ability to do so persistently.

Gene activity, chromatin accessibility and DNA methylation are influenced by promoter GC content (39–41). We therefore examined the contribution of GC content to the difference between persistent silencing and re-activation at target regions. There were no significant differences in GC density and the number of CpG dinucleotides in the 250 bp target regions (Supplementary Figure S4A). We extended our analysis to CpG islands (CGI) that overlapped the target region by at least 1 base pair. Three target promoters (*ZNF274, STAT5A, EPCAM*) did not overlap with their respective CpG island and were not included in this analysis. There were also no significant differences in the length of CGIs, number of CpGs or CpG percentage in the CGI (Supplementary Figure S4A and Supplementary Table S3).

Recent studies have combined the same epigenetic editing domains (KRAB and DNMT3A + L) onto one dCas9 molecule. Persistent silencing has been improved by using different promoters, different linker lengths and fusion to either the N- or C-terminus of dCas9 (27,28). The highest performing version by Nuñez *et al.* (28) was DNMT3A-3L-dCas9-KRAB (CRISPRoff v2) and was reported to persistently silence many endogenous loci. We wanted to compare our KRAB/D3A + L epi-dCas9 to CRISPRoff v2 at 5 target genes using our gRNAs (Supplementary Figure S4B). We observed various degrees of persistent silencing for both epigenetic editing platforms. Out of 5 genes tested, we observed similar repression at one gene (*FMR1*) and stronger repression with the epi-dCas9 system at one gene (*PLAGL1*) while CRISPRoff v2 showed more persistent silencing at the remaining three genes (*FOSL1, EPCAM, OTX1*). In summary, the efficiency of engineering persistent silencing is gene-specific, and we have yet to uncover predictive regulatory features.

### Distinct *cis* chromatin features correlate with short- and long-term silencing, particularly H3K27ac predicting resistance to persistent silencing

To gain insight into the principles of engineering persistent silencing, we systematically analyzed the relationship of gene silencing with underlying chromatin features. Actively transcribed promoters are decorated with active histone marks H3K4me3 and H3K27ac. Although promoters in this study carry both histone marks in addition to DNaseI hypersensitivity sites (Figure 1B), they responded differently to epigenetic intervention using KRAB/D3A + L epi-dCas9. We hypothesized that underlying chromatin features could at least in part account for these differences. We integrated ENCODE epigenome data with target gene silencing to identify chromatin features that are permissive or resilient to persistent silencing. Enrichment scores of histone marks and other chromatin factors were obtained from ChIP-seq data sets in K562 cells in addition to whole genome bisulfite data for DNA methylation (6,42) (Supplementary Table S2). Average enrichment scores at the 250-bp target regions were then correlated with short- and long-term target gene expression using Spearman correlation and significance was evaluated after Benjamini–Hochberg multiple-testing corrections (Supplementary Figures S5 and S6). The ability of KRAB/D3A + L epi-dCas9 to engineer long-term silencing inversely correlated with H3K27ac at the 250-bp target region (Spearman correlation *r =* 0.7*, q* = 0.005, Figure 2D). Relative H3K27ac enrichment ranged from 5.5 to 30.2 (median 15) with exception of *FOSL1* which was an outlier with a mean H3K27ac enrichment score of 64 (Supplementary Table S3). To ensure that correlations are not driven by one or two outliers we conducted a subsampling analysis. We confirmed that correlations remained significant after removal of any two data points (Supplementary Figure S7). Active promoters are decorated with H3K4me3 and H3K9ac in addition to H3K27ac (43) but only H3K27ac correlated with re-activation of short-term repressed genes. The promoter marks H3K4me3 and H3K9ac showed no correlation with amenability for persistent silencing (Spearman correlation *q* = 0.5 and *q =*0.3, respectively). We then tested KRAB/DNMT3A + L for its ability of to elicit long-term silencing at a subset of target genes in HeLa S3, another ENCODE cell line. Overall, higher level of persistent silencing was attained in HeLa S3 cells compared to K562 cells (Supplementary Figure S8). H3K27ac levels were similar to K562 cells at most genes (Spearman correlation *P* = 0.002, Supplementary Figure S8A, Supplementary Table S3). *FOSL1* had the biggest difference with a H3K27ac enrichment score of 15 in HeLa S3 cells compared to 64 in K562 cells. KRAB/D3L + L epi-dCas9 was able to persistently silence *FOSL1* in HeLa S3 cells, but not in K562 cells (Supplementary Figure S8B). Together, these data indicate that H3K27ac at promoters contributes to gene-specific differences in the ability to transition from an active into a silenced chromatin state.

While binding events of transcription factors are localized to narrow chromatin regions, modified histones spread over larger regions encompassing multiple nucleosomes. We therefore expanded our analysis to 1 kb flanking the target region (250 bp ± 1 kb; Figure 2E, Supplementary Figure S5B). The expanded region was expected to better capture chromatin features localizing further upstream or downstream of the transcription start site (TSS). Indeed, the extended target region identified RNA polymerase POLR2A (Spearman correlation *r* = 0.6*, q* = 0.015) and CTCF (Spearman correlation *r* = 0.6, *q* = 0.015) in addition to H3K27ac (Spearman correlation *r =*0.6*, q* = 0.03) as chromatin factors that correlated with reactivation and restoration to baseline expression levels (Figure 2E).

We then investigated if the same or different chromatin features correlate with short-term expression levels affected by KRAB/DNMT3A + L. The results showed that H3K27ac, POLR2A and CTCF did not correlate with ability to elicit short-term repression and were unique to persistent silencing (Figure 2E and Supplementary Figure S5). Conversely, H3K36me3 enrichment was indicative of reduced ability of KRAB/D3A + L epi-dCas9 to induce short-term repression (Spearman correlation *r* = 0.4*, q* = 0.014, Figure 2F). We only observed this correlation in the expanded region (250 bp ± 1 kb), probably because H3K36me3 is associated with active transcription in gene bodies and is hence more prevalent downstream of TSS (4). This corroborates our previous observation that genes with high baseline expression (FPKM) resist short-term repression (Refractory genes; Figure 1F). Several other features did not significantly correlate with either short- or long-term silencing, including pre-existing DNA methylation (Supplementary Figure S5 and S6). Since CpG islands (CGIs) are usually not methylated, we also looked at methylation levels excluding CGI (outside CGI) and methylation of the CGI itself (inside CGI). We did not observe significant correlation between baseline DNA methylation and silencing ability.

Pre-existing chromatin features may not only be indicative of ability to engineer silencing but could also influence the ability to elicit DNA methylation at the target promoter. Indeed, we found a significant inverse correlation between baseline H3K27ac and long-term DNA methylation increase facilitated by KRAB/D3A + L epi-dCas9 (Spearman correlation *r = -*0.82*, q* = 0.01; Figure 2G).

In summary we identified two distinct epigenetic signatures associated with either short- or long-term repression by KRAB/D3A + L epi-dCas9. Short-term repression correlated with features of active transcription, while resistance to persistent silencing correlated with H3K27ac, CTCF and RNA polymerase enrichment, supporting the importance of these marks for cellular identity (Figure 2F). In addition, H3K27 acetylation also showed a negative correlation with DNA methylation increase at the target locus. This suggests that H3K27 acetylation influences heritable silencing by modulating different levels of DNA methylation (Figure 2H).

### KRAB/D3A + L and Ezh2/D3A + L efficiently initiate short- and long-term repression, but show gene-specific differences

**Figure 3.**
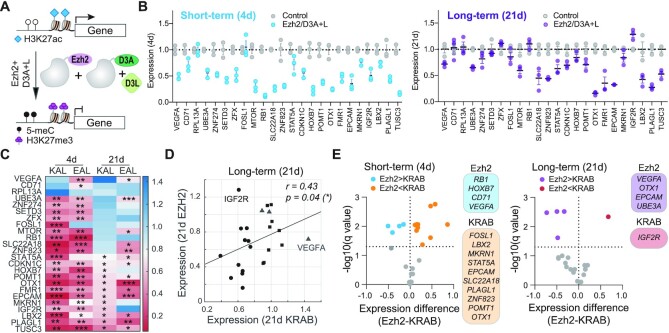
KRAB/D3A + L and Ezh2/D3A + L efficiently initiate short-term repression and long-term silencing, but show gene-specific differences. (**A**) Cartoon depicts Ezh2/D3A + L epi-dCas9 highlighting changes of epigenetic marks and gene expression. (**B**) Short- and long-term expression was evaluated by RT-qPCR (*n* = 3; multiple unpaired *t*-tests with Benjamini–Hochberg correction). Relative expression has been normalized to *GAPDH* and to control transfection with non-targeting gRNA. (**C**) Heatmap of short- and long-term target gene expression comparing KRAB/D3A + L (KAL) and Ezh2/D3A + L (EAL) epi-dCas9 targeting platforms. Genes are arranged in rows and treatment parameters in columns. The *q*-values (*n* = 3; multiple unpaired *t*-tests with Benjamini–Hochberg correction) are relative to control treatment and are indicated inside the heatmap (*q< 0.05, **q< 0.01 and ***q< 0.001). Red color correlates with silenced (lowest expression) and blue color with re-activated (highest expression) genes. (**D**) Scatter plot depicting Spearman correlation of long-term expression elicited by Ezh2- and KRAB-based epi-dCas9 platforms. (**E**) Volcano plots evaluating differential short-term gene repression and long-term silencing capacity of both epi-dCas9 platforms. Difference in relative expression (KRAB-Ezh2) is plotted on the *x*-axis. Negative values identify genes that are more efficiently silenced by Ezh2/D3A + L epi-dCas9, while positive values identify genes that are more efficiently silenced by KRAB/D3A + L epi-dCas9. The horizontal dotted line indicates *q =*0.05 on the *y*-axis of volcano plots.

Epigenetic effectors KRAB and Ezh2 offer complementary capacity in engineering long-term silencing (25,29). While KRAB recruits complexes to deposit H3K9me3 and DNA methylation to form constitutive heterochromatin, Ezh2 deposits H3K27me3 and facilitates formation of facultative heterochromatin through recruitment of the PRC2 complex. We previously demonstrated that targeting PRC2 (Ezh2-dCas9) and DNA methyltransferases (DNMT3A-dCas9 + DNMT3L) to the *HER2* promoter deposits H3K27 methylation and can overcome re-activation allowed by KRAB (25). Indeed, targeting *HER2* but replacing KRAB-dCas9 with Ezh2-dCas9 produced much greater long-term silencing. In this study, we wanted to investigate if the observation at a single gene (*HER2*) is generalizable to other genes. In particular, we wanted to test the ability of Ezh2/D3A + L epi-dCas9 to elicit persistent silencing where KRAB/D3A + L epi-dCas9 combination performed poorly. Therefore, we targeted the same 24 genes, but substituted KRAB-dCas9 for Ezh2-dCas9 (Figure 3A). Gene expression levels were measured by RT-qPCR 4d (short-term) and 21d (long-term) after transfection and compared to the control (no gRNA) (Supplementary Table S3). *RPL13A, FOSL1* and *STAT5A* were refractory to Ezh2/D3A + L, while *VEGFA, CD71* and *RPL13A* were refractory to repression by KRAB/D3A + L, highlighting the mechanistic differences between these two epigenetic editing systems (Figure 3B and C). Both KRAB and Ezh2 epi-dCas9 efficiently initiated short-term repression but in a gene-specific manner (Spearman correlation, *P =*0.06). 14 of the 21 short-term repressed genes showed persistent silencing after Ezh2/D3A + L treatment. Although Ezh2/D3A + L and KRAB/D3A + L had overall similar ability to engineer long-term silencing (Spearman correlation *P =*0.04; Figure 3D), both treatments showed gene-specific differences (Figure 3E). Ezh2/D3A + L engineered persistent silencing of *VEGFA* and *UBE3A*, which were resistant to silencing by KRAB/D3A + L. On the other hand, Ezh2/D3A + L failed to elicit long-term repression of *IGF2R*, a gene that was persistently silenced by KRAB/D3A + L. In addition, some genes were differentially repressed. For example, Ezh2/D3A + L enabled more efficient long-term silencing of *EPCAM* and *OTX1* when compared to KRAB/D3A + L (2- and 4-fold, respectively).

In summary, KRAB/D3A + L and Ezh2/D3A + L efficiently initiated short- and long-term repression at a subset of target genes but showed gene-specific differences.

### Ezh2/D3A + L epi-dCas9 initiates robust DNA methylation and elicits persistent silencing irrespective of target site H3K27ac enrichment

**Figure 4.**
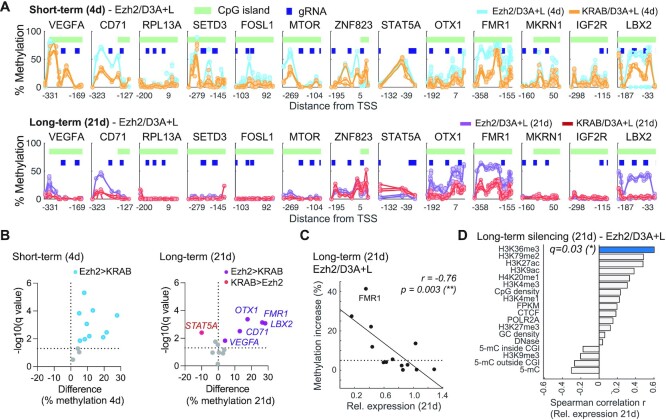
Ezh2/D3A + L epi-dCas9 elicits robust and stable DNA methylation and overcomes dependency on H3K27ac observed with KRAB/D3A + 3L. (**A**) Methylation profiles comparing the percent increase over baseline methylation (*n* = 3) after treatment with KRAB/D3A + L and Ezh2/D3A + L epi-dCas9 epigenetic editing platforms. Short-term effects are shown in the top panel and long-term in the bottom panel. Each circle represents a CpG, pale green bars represent CpG islands and blue boxes depict gRNA target sites. (**B**) Volcano plots highlighting statistical significance of differential methylation facilitated by either KRAB-dCas9 or Ezh2-dCas9 in presence of targeted DNA methylation. Positive values identify genes that are more efficiently methylated by Ezh2/D3A + L epi-dCas9, while negative values identify genes that are more efficiently silenced by KRAB/D3A + L epi-dCas9. The horizontal dotted line indicates *q*= 0.05 on the *y*-axis of volcano plots. (**C**) Scatterplot shows Spearman correlation *r* of mean methylation increase with long-term silencing (21 d). A horizontal dotted line indicates a 5% methylation increase. (**D**) Correlative analysis of long-term silencing by Ezh2/D3A + L epi-dCas9 with enrichment of chromatin features in the 250-bp target region (*q< 0.05; Spearman correlation with Benjamini–Hochberg correction).

The relationship of DNA methylation and H3K27me3 deposition by Ezh2 is not understood. In fact, H3K27me3 and DNA methylation are repressive marks that usually do not co-localize. We used this opportunity to investigate how targeting Ezh2 in combination with DNA methyltransferases influences deposition and stability of *de novo* DNA methylation at target promoters (Supplementary Figure S9A and Supplementary Table S3). Intriguingly, Ezh2/D3A + L epi-dCas9 initiated DNA methylation at all tested gene promoters with a methylation increase ranging from 11 to 52% (Supplementary Figure S9B). In comparison, methylation increase by KRAB/D3A + L ranged from 2 to 27%. The three genes that were refractory to Ezh2/D3A + L treatment showed a methylation increase of 12, 14 and 27% for *RPL13A*, *FOSL1* and *STAT5A*, respectively. Most importantly, Ezh2/D3A + L elicited higher *de novo* short-term DNA methylation at 10 of the 13 genes tested when compared to KRAB/D3A + L (Figure 4A and B). The remaining three genes (*ZNF823, STAT5A, IGF2R*) were methylated to comparable levels between the two treatments. We then evaluated long-term methylation levels and observed an overall decrease in DNA methylation with the exception of *OTX1* (Figure 4B, Supplementary Figure S9A and C). Methylation increase reverted to baseline levels (<5% methylation increase) for *RPL13A, FOSL1* and *IGF2R* but remained above 10% for 6 genes. KRAB/D3A + L was able to maintain higher methylation levels at one of the genes (*STAT5A*; 16%) compared to Ezh2/D3A + L (6%, Figure 4B). *FMR1, OTX1* and *LBX2* were persistently repressed by both Ezh2 and KRAB-based epi-dCas9 combinations, but DNA methylation maintained after Ezh2/D3A + L treatment was not only stable, but surpassed methylation levels elicited by KRAB/D3A + L. Although methylation of *LBX2* by KRAB/D3A + L was almost completely lost by day 21, *LBX2* remained silenced. DNA methylation and gene silencing mediated by KRAB/D3A + L showed poor correlation (Spearman correlation *P =*0.5 and 0.09 for short-term and long-term silencing, respectively; Supplementary Figure S2E). Similarly, methylation increase and relative short-term expression at 4 days did not correlate upon Ezh2/D3A + L epi-dCas9 treatment (Spearman correlation *r = -*0.19*, P* = 0.5). However, the enhanced methylation increase by Ezh2/D3A + L at 21 days showed strong correlation with persistent silencing (Spearman correlation *r = -*0.76*, P* = 0.002; Figure 4C). We note that we did observe outliers. For example, Ezh2/D3A + L was able to maintain a methylation increase at *VEGFA* and *CD71* (10 and 28%, respectively), but only *VEGFA* was persistently silenced while *CD71* was re-activated. Our data demonstrate that although H3K27me3 and DNA methylation do not typically colocalize, co-recruitment of Ezh2 (responsible for H3K27me3 deposition) and DNA methyltransferases created an environment permissive to more robust *de novo* and stable DNA methylation at a subset of genes.

We again performed read-level methylation analysis to investigate distribution of methylation across cells. Methylation profiles varied depending on target region. Four days after treatment with Ezh2/D3A + L epi-dCas9, three genes (*VEGFA, CD71* and *SETD3*) displayed unimodal distribution with a large shift in DNA methylation (mode: 30%, 44% and 29%) compared to 0–1% methylation in control cells (Supplementary Figure S10A). Interestingly, over time methylation of these three genes followed different trajectories. At 21 days after transfection, *VEGFA* and *SETD3* showed unimodal distributions with the majority of reads being unmethylated. *VEGFA* showed long-term silencing while *SETD3* was reactivated to baseline levels by 21 days. On the other hand, *CD71* showed a bimodal distribution after 21 days suggesting that two cell populations have formed, one mostly unmethylated (42% of reads) and a second population (58% of reads) with an average of 26% methylation. Methylation was not sufficient to maintain long-term silencing of *CD71*. For two other genes (*FMR1* and *LBX2*), bimodal distribution was already established 4 days after transfection, with 78% and 67% of the reads being highly methylated (distribution mode: 77% and 74%). This distribution remained stable and was also observed at 21 days (Supplementary Figure S10). *FMR1* and *LBX2* showed long-term silencing. These data demonstrate that different loci not only have different amenability to long-term silencing but also vary in response to engineering long-term methylation.

We next asked the question if H3K27 tri-methylation is required for the DNA methylation increase with Ezh2/D3A + L epi-dCas9. We performed ChIP-qPCR for two genes that were reactivated (*SETD3, IGF2R*) after initial short-term repression by Ezh2/D3A + L epi-dCas9 treatment. We detected a temporary H3K27me3 increase at *SETD3*, while no H3K27me3 increase was detected at *IGF2R* promoter relative to untreated control cells (Supplementary Figure S11A). We also assessed H3K27me3 deposition at two genes that were persistently silenced (*OTX1, PLAGL1*; Supplementary Figure S11A). *OTX1*, which became heavily methylated (27.4% 5-mC increase at 21 days), displayed a temporary H3K27me3 increase at 4 days that was lost by 21 days. However, no H3K27me3 increase was detected at the *PLAGL1* promoter. Temporary H3K27me3 deposition may contribute to increase in DNA methylation and persistent silencing at a subset of genes. We further evaluated histone deacetylation of H3K27ac at *SETD3* (re-activated gene) and three persistently silenced genes (*OTX1, FMR1, PLAGL1)* after treatment with KRAB/D3A + L or Ezh2/D3A + L epi-dCas9. There was no reduction of H3K27ac at the reactivated *SETD3* gene while H3K27ac reduction for the three silenced genes was persistent and comparable for both treatments (Supplementary Figure S11B).

We then investigated if particular chromatin features correlate with the ability of Ezh2/D3A + L epi-dCas9 to persistently repress target genes. H3K36me3, the mark of elongation and active transcription, was the only chromatin feature identified. H3K36me3 correlated with resistance to persistent silencing at 21 days in the 250 bp target region or the +/- 1kb expanded target region (Spearman correlation *q* = 0.03 and 0.04, respectively, Figure 4D, Supplementary Figure S11C and D). This is contrary to persistent silencing by KRAB-based epi-dCas9 that inversely correlated with H3K27ac. We did not detect a relationship between H3K27ac and persistent repression by Ezh2/D3A + L at the 250-bp target region (Spearman correlation *q* = 0.1). This is distinctly different from the correlation observed with KRAB/D3A + L (Spearman correlation *q* = 0.005) and offers a glimpse at the mechanistic differences between these two epigenetic editing platforms.

### Long-term silencing by KRAB/D3A + L, but not Ezh2/D3A + L, corresponds with divergence of epigenetic states in different cell types

**Figure 5.**
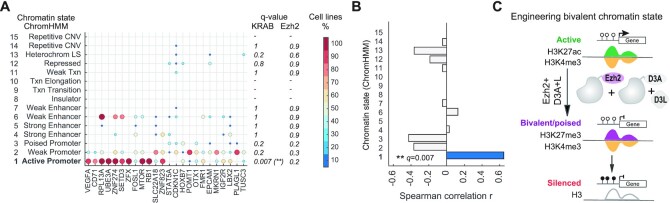
Active promoter chromatin state across different cell types correlates with resistance to persistent silencing by KRAB/D3A + L, but not Ezh2/D3A + L epi-dCas9. (**A**) Summary statistic of ChromHMM 15-chromatin state model of 9 histone marks in 9 cell lines at 24 target genes. For each of the 15 chromHMM states (rows) and 24 regions (columns), the color and size of the circle represents frequency at which the chromatin state is observed across 9 cell types in the target region. For instance, *MTOR* target region contains state 1 in 9/9 = 100% cell lines, state 2 in 5/9 = 56% cell lines, and state 6 in 3/9 = 33% cell lines. The columns are sorted by the relative expression at 21 d for KRAB/D3A + L. The *q*-value columns show statistical significance of Spearman correlation of this frequency and the relative expression at 21 d for KRAB/D3A + L and Ezh2/D3A + L, adjusted using Benjamini–Hochberg correction (**q< 0.01). (**B**) Bar graph depicting Spearman correlation for 15 ChromHMM chromatin states (**q< 0.01; Spearman correlation with Benjamini–Hochberg correction) of state frequency across cell lines with relative expression at 21d for KRAB/D3A + L. (**C**) Proposed model for engineering of a transient bivalent chromatin state by Ezh2/D3A + L as indicated by simultaneous display of active (H3K4me3) and repressive (H3K27me3) chromatin marks. Genes with bivalent signature are repressed. Bivalent signature pre-disposes the target locus to persistent silencing by DNA hypermethylation.

Gene expression changes of individual genes are part of normal development and cell differentiation. As a result, different sets of genes are expressed at different time points and/or different cell types. ChromHMM captures combinatorial patterns of histone modifications identifying different gene expression states and functional genomic regions across different cell types and tissues (5,44,45). Therefore, ChromHMM is a valuable resource to evaluate genomic regions that transition between different chromatin states depending on cell type. We applied the 15-core chromatin state model that was derived from nine histone marks across nine cell lines (37). We hypothesized that genes that are expressed in one cell type but silenced in another could be more amenable to engineering persistent silencing. When we evaluated chromatin states of our 24 target regions across nine cell lines (Figure 5A and B, Supplementary Figure S11A), an active promoter state across multiple cell lines inversely correlated with persistent silencing at 21 days (Spearman correlation *q =*0.007). This supports the hypothesis that promoters that display an active promoter state across multiple cell types are less amenable to persistent silencing by KRAB/D3A + L epi-dCas9. On the other hand, amenability to persistent silencing corresponded with alternative chromatin states in other cell types, such as weak and poised promoters, repressed chromatin and heterochromatin (Figure 5B). For example, we observed heterochromatin state at the *CDKN1C* promoter in HepG2 cells and repressed chromatin state at the *STAT5A* promoter in HMEC cells. As another example, the *STAT5A* promoter was also associated with a poised promoter state in HepG2 and NHEK cells. The poised (bivalent) promoter state is marked by the presence of the active mark H3K4me3 and the repressive mark H3K27me3. Genes with a bivalent signature are not expressed. KRAB/D3A + L epi-dCas9 enabled persistent silencing at gene promoters with divergent epigenetic profiles, but not at promoters with an active promoter chromatin state across multiple cell types (Figure 5A).

We then asked the question if persistent silencing by Ezh2/D3A + L epi-dCas9 shows the same dependencies on divergent chromatin states. Interestingly, there was no correlation of any state with ability to elicit long-term silencing (Figure 5A and Supplementary Figure S12B), adding to the evidence that KRAB and Ezh2 offer complementary silencing platforms.

Taken together, we were able to identify distinct chromatin features associated with long-term silencing by KRAB/D3A + L epi-dCas9. Interestingly, H3K27ac enrichment at the target region and a consistent active promoter state across different cell types counteract the ability to elicit persistent silencing by KRAB/D3A + L, but not Ezh2/D3A + L.

## DISCUSSION

Epigenetic editing tools offer a fantastic opportunity to study chromatin biology, especially causal relationships between epigenetics and expression. Understanding underlying mechanisms and pathways of cell state transitions will help create programmable and reversible epigenome editing platforms for precision medicine. After transient expression of a combination of epigenetic editors, we and others have observed gene-specific variability of desired long-term effects at a handful of loci (23,25,27,28). We took a candidate approach to elucidate some of the pre-existing chromatin features that could potentially discern amenability of a locus to persistent epigenetic editing.

Our study aimed at evaluating the relationship between epigenetics and target gene expression while at the same time minimizing CRISPRi effects. As the name implies, CRISPR interference (CRISPRi) functions by interfering with transcription by blocking polymerase binding (21,22,46). Most effective interference was observed when targeting CRISPR/dCas9 without any effector domain or fused to a repressor domain such as KRAB to the window of + 25 to + 75 nucleotides immediately downstream of the transcriptional start site (21,47,48). The principle of CRISPRi has been used to transiently reduce transcription at specific target sites as well as in genome-wide screens (47,48). Interestingly, by simply targeting dCas9-KRAB to the proximal promoter, contributions of the KRAB transcriptional repressor domain could be distinguished from that of dCas9 which did not show CRISPRi effects upstream of the TSS (22). A recent study identified the targeting window for heritable epigenetic editing to be much broader spanning 1 kb centered around the TSS (28). We therefore designed gRNAs in our study to the target window of -1 to -250 nucleotides upstream of the TSS to minimize overlap with the CRISPRi target region. It is plausible that targeting combinations of epigenetic editors, such as used in our study, to the window downstream of TSS and interfering with transcription would have resulted in higher short- and long-term repression efficiency, but analysis of direct epigenetic effects would have been much more convoluted.

After transient treatment with targeted transcriptional repressors (KRAB-dCas9 or Ezh2-dCas9) and targeted DNA methylation (DNMT3A-dCas9 + DNMT3L) we performed correlation analysis with baseline expression, genomic features, DNA methylation and 12 different chromatin features (Supplementary Table S2). We identified pre-existing chromatin features that distinctly associated with long-term silencing. Resistance to long-term silencing by KRAB-dCas9/dCas9-D3A + L in particular was associated with H3K27ac, RNA polymerase POL2RA and CTCF occupancy. This is intriguing since H3K27ac has been widely studied in association with active enhancers, but a functional role of H3K27ac at promoters (apart from an open chromatin feature) has not yet been elucidated. Our data suggest that H3K27ac is a key feature associated with resisting cell state transitions to inactive chromatin and protecting the target gene from persistent silencing. We discovered a strong inverse correlation between H3K27 acetylation and engineered DNA methylation that could be the underlying reason for differential ability to elicit persistent silencing. It remains to be seen if pre-treatment with targeted histone deacetylases (HDACs) and removal of H3K27 acetylation can enhance engineered promoter methylation and long-term silencing. Our study supports the possibility that promoters (similar to enhancers) contain information about the current and future developmental potential of a cell, as well as its ability to respond to external cues. Future studies will aim to shed light on the network of chromatin regulators assisting H3K27ac in this role.

When replacing KRAB-dCas9 with Ezh2-dCas9, we no longer observed the inverse relationship between engineering epigenetic memory and H3K27ac enrichment. The H3K27 methyltransferase Ezh2 catalyzes H3K27 tri-methylation and recruits the PRC2 repression complex (49). It is hence not surprising that engineering epigenetic memory by co-targeting Ezh2-dCas9 can overcome H3K27ac-mediated resistance to silencing. The knowledge that H3K27 acetylation is one of the features that underlies gene-specific variability, can inform future adjustments to help engineer gene-specific and cell-specific epigenetic memory.

In nature, bivalent genes are poised (paused) to quickly respond to developmental cues. This unique class of genes consist of promoters that contain both, active (H3K4me3) and repressive (H3K27me3) marks (7,50,51). Bivalent genes are prevalent in pluripotent stem cells, but to a lesser extent in other cell types. During development, these genes can transition to either a repressed or active chromatin state. Bivalent promoters are occupied by Ezh2, the H3K27 histone methyltransferase of the Polycomb group repressor complex (PRC2) (52). Epigenetic editing with Ezh2-dCas9/DNMT3A + L tri-methylates H3K27 (25,29) in promoters that already contain the active mark H3K4me3, thereby enabling a transient repressed bivalent state and facilitating the transition to a long-term silenced state (Figure 5C). Engineering a bivalent chromatin state presents new opportunities to investigate chromatin and regulatory dynamics at endogenous loci or synthetic circuits.

Intriguingly, genes with a bivalent signature in stem cells are predisposed to become heritably silenced by DNA methylation (53). Persistent methylation of bivalent genes was induced by either direct targeting of promoter regions with DNA methyltransferase DNMT3A (54) or by overexpression of its enhancer protein DNMT3L (55). It is worth noting that targeting DNMT3A to CpG island promoters elicited transient methylation at most promoters, but methylation was stable only at promoters deemed bivalent in ES cells (54). Moreover, aberrant hypermethylation of bivalent chromatin is also observed in cancer (56–59). On the other hand, TET enzymes protect bivalent genes from becoming methylated (60–62). Dioxygenase TET1/2 and 3 lead to conversion of 5-mC to 5-hydroxymethylcytosine (5-hmC) that can further be demethylated. Knock-out of all three TET isoforms leads to prominent hypermethylation of bivalent promoters (60). Changes of DNMT3A and TET1 influence PRC2 complex activity, indicating a dynamic relationship (60–62). Although the detailed mechanisms remain elusive, there is an undeniable relationship between DNMT3A, TET1 and H3K27me3 and/or PRC2, especially in the context of bivalent chromatin. Precise spatiotemporal expression of our epigenetic editor combinations offers the unique opportunity to further investigate dynamic and causal relationships during normal and cancerous development.

As evidence of these relationships, we have observed robust and stable DNA methylation at target genes. In this study, we demonstrate that co-recruitment of Ezh2 with DNA methyltransferases manifests in stronger bursts of DNA methylation at 77% (10 out of 13) loci tested when compared to the targeted KRAB/DNA methyltransferase approach. More stable and robust long-term DNA methylation was observed at 5 out of 13 genes with the Ezh2-based approach, but only at one gene with the KRAB-based approach. Higher levels in DNA methylation elicited by Ezh2/D3A + L epi-dCas9 correlated well with the ability to maintain persistent silencing. This suggests that higher DNA methylation levels are important for maintenance of DNA methylation and maintenance of a repressive epigenetic state. In fact, our previous work at the *HER2* locus in HCT116 cells has shown that DNA methylation obtained with Ezh2-dCas9/dCas9-DNMT3A + L was not only more robust but was also able to spread over >1 kb (25).

Taken together, precise targeting by KRAB- or Ezh2-dCas9 and DNA methyltransferase offer two alternative platforms for engineering epigenetic memory. Both epigenetic editing platforms operate with different underlying mechanisms that can be elucidated using these targeting systems. While our study design does not allow for study of kinetics, individual epigenetic silencing domains follow distinct dynamic trajectories using a synthetic circuit (24). The KRAB repressor domain and histone deacetylase HDAC4 act quickly reaching maximum repression within hours, while DNA methyltransferase B (DNMT3B) reaches highest repression between 40 and 60 h. Similarly, different epigenetic silencing domains had different outcomes in terms of epigenetic memory. While HDAC4 imparted only short-term repression that was lost after 5 days, DNMT3B was able to maintain long-term silencing for the 30-day period tested (24). These exciting single-cell level observations are based on expression from an artificial chromosome. Future studies will help elucidate dynamics and dependencies of epi-dCas9 combinations at endogenous genomic regions.

In conclusion, our candidate gene approach gave us a glimpse into chromatin features correlated with transition to a repressed state and persistent silencing. The information gained in this study will advance the capabilities of us and others to create targeted persistent epigenetic changes for the study and treatment of disease, and also provide fundamental insights into understanding contextual cues to more predictably engineer persistent silencing at a given locus.

## DATA AVAILABILITY

All plasmids used in this study are available on Addgene. Genome-wide datasets used in this study are listed in Supplementary Table S2. All data that led to the conclusions of this study are available in Supplementary Figures and Tables.

## Supplementary Material

gkac123_Supplemental_Files
