# Supplementary material for: Determinants of heritable gene silencing for KRAB-dCas9 + DNMT3 and Ezh2-dCas9 + DNMT3 hit-and-run epigenome editing
Source: Nucleic Acids Res. 2022 Mar 2;50(6):3239–53. doi: 10.1093/nar/gkac123 (PMC8989539; doi:10.1093/nar/gkac123)
Supplement: gkac123_Supplemental_Files [file gkac123_supplemental_files.zip › SUPPL_combo.pdf]

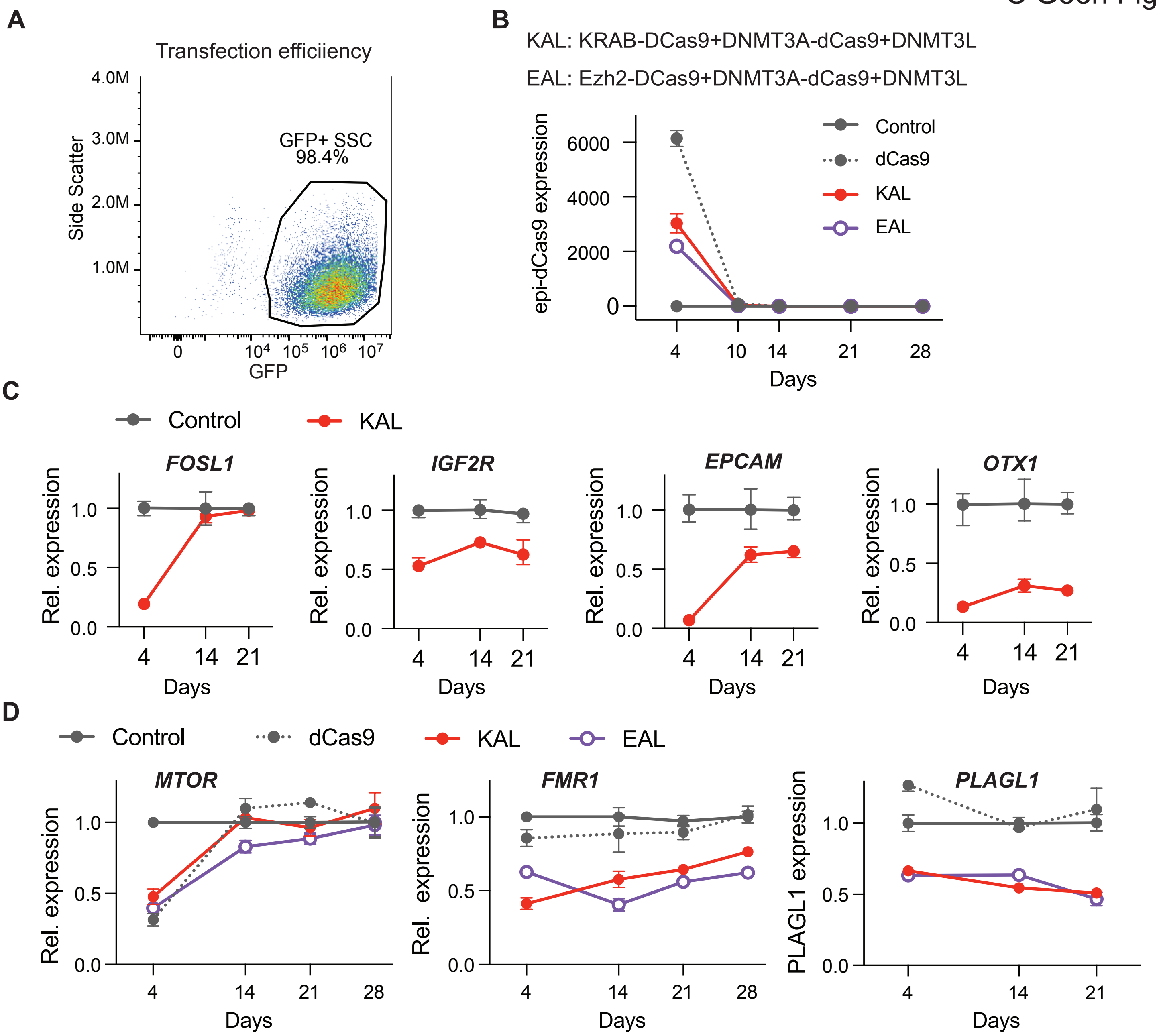

Figure S1. Time course of epi-dCas9 expression and long-term target gene silencing. A. Representative FACS analysis of K562 cells 72 hours after transfection with the GFP reporter plasmid pMax-GFP. Transfection efficiencies were routinely >95%. **B.** Expression of epi-dCas9 was evaluated by RT-qPCR over a time course of 28 days (n=3, error bar is +/- SEM). KRAB/D3A+L (KAL) and Ezh2/D3A+L (EAL) epi-dCas9 expression was compared to dCas9 alone (without epigenetic editor domain) and to cells transfected with a control plasmid (pMax-GFP). Gene expression has been normalized to GAPDH. Epi-dCas9 expression rapidly dropped and returned to baseline by 10 days after transfection. Different expression levels were observed (dCas9>KAL>EAL). **C.** Stability of heritable target gene silencing was determined by RT-qPCR 14 and 21 days after co-transfection of plasmids expressing KRAB/D3A+L (KAL) epi-dCas9 and 4 targeting gRNAs (n=3, error bar is +/- SEM). Gene expression has been normalized to *GAPDH* and is compared to the control without targeting gRNAs. Relative expression levels have stabilized by 14 days and did not increase further by 21 days. **D.** Stability of heritable target gene silencing was determined by RT-qPCR over a 28 day time course as described in (C). Long-term silencing was evaluated for KRAB/D3A+L (KAL) and Ezh2/D3A+L (EAL) and was compared to a control without gRNAs and to dCas9 without epigenetic effector domains. dCas9 by itself was unable to elicit long-term silencing. In one case dCas9 by itself did cause temporary repression as is known to occur for CRISPRi.

O’Geen Fig.S2

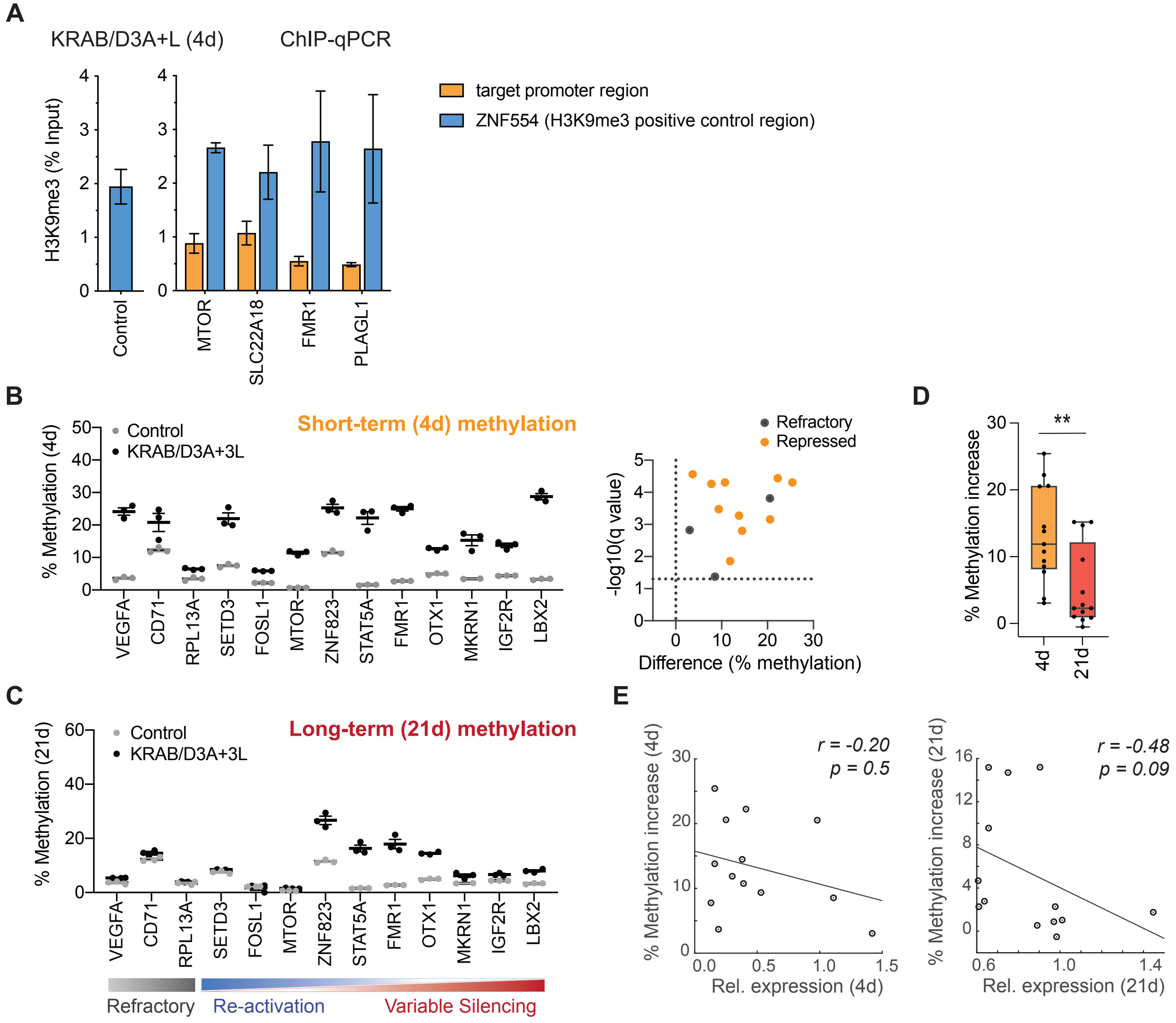

Figure S2. Engineered DNA or histone methylation do not predict long-term silencing by KRAB/D3A+L epi-dCas9. A. Chromatin immunoprecipitation followed by qPCR (ChIP-qPCR) analysis for H3K9me3 of untreated control cells and cells treated with KRAB/D3A+L epi-dCas9 for 4 days. Targeted promoters are indicated on the x-axis. H3K9me3 ChIP enrichment is plotted as % input on the y-axis for the target promoter region and a positive control region *ZNF554* (mean +/- SEM, n=2-3). **B.** Methylation has been measured for 13 genes using next-gen bisulfite amplicon sequencing. % methylation (n=3 biological replicates; mean +/- SEM) is plotted on y-axis for untreated control cells (baseline level) and is compared at **(B)** short-term (4d) and **(C)** long-term (21d) time points. Volcano plot shows significant changes in DNA methylation. The horizontal dotted line crosses the y-axis at $q=0.05$. “Refractory” and “Repressed” refer to categories defined in Figure 1. **D.** Box plot comparing mean methylation increase of silenced and re-activated genes ($**p<0.01$). **E.** Scatter plots visualize correlation between methylation increase and relative gene expression at short and long-term time points (Spearman correlation with Benjamini-Hochberg correction).

O’Geen Fig.S3

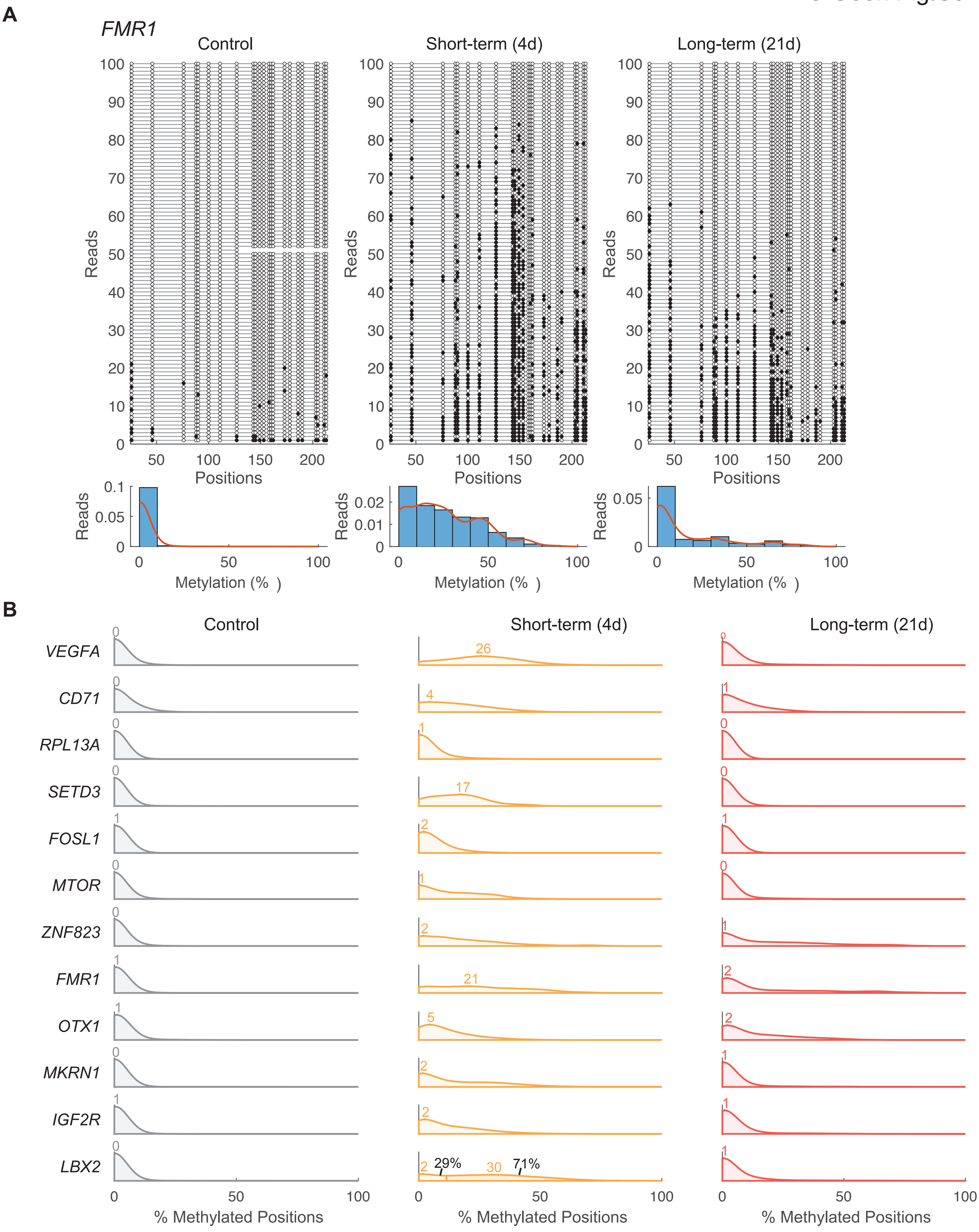
A
FMR1
Control
Short-term (4d)
Long-term (21d)
Reads
Positions
Metylation (%)
B
Control
Short-term (4d)
Long-term (21d)
VEGFA
CD71
RPL13A
SETD3
FOSL1
MTOR
ZNF823
FMR1
OTX1
MKRN1
IGF2R
LBX2
29%
71%
% Methylated Positions

Figure S3. Read-level methylation analysis shows unimodal distribution after KRAB/D3A+L epi-dCas9 treatment. A. Read-level methylation analysis is shown for the *FMR1* promoter region in untreated control cells, 4 days and 21 days after KRAB/D3A+L epi-dCas9 treatment. Lollipop plots depict methylation status of individual sequence reads derived from targeted bisulfite sequencing data. Black circles represent methylated CpGs, while open circles are unmethylated CpGs. 100 representative reads are graphed against CpG positions and the reads are shown sorted according to their average methylation value. For each read, the percentage of methylated positions was computed and their frequency distribution plotted in the histogram below. A curve was fitted using ksdensity. **B.** The ksdensity distribution of % methylated positions across reads is plotted for all 12 regions. Y-axis (probability density function across reads) has been set to the same range for all plots (control, 4d, 12d). To determine unimodal or bimodal distributions, peaks and valleys were computed using findpeaks. In this visualization, data from three replicates are pooled together. The black numbers represent percentage of reads in the two modes of the distribution (in bimodal distributions only). The colored numbers represent the mode of the distribution (position of the peak).

O'Geen Fig.S4

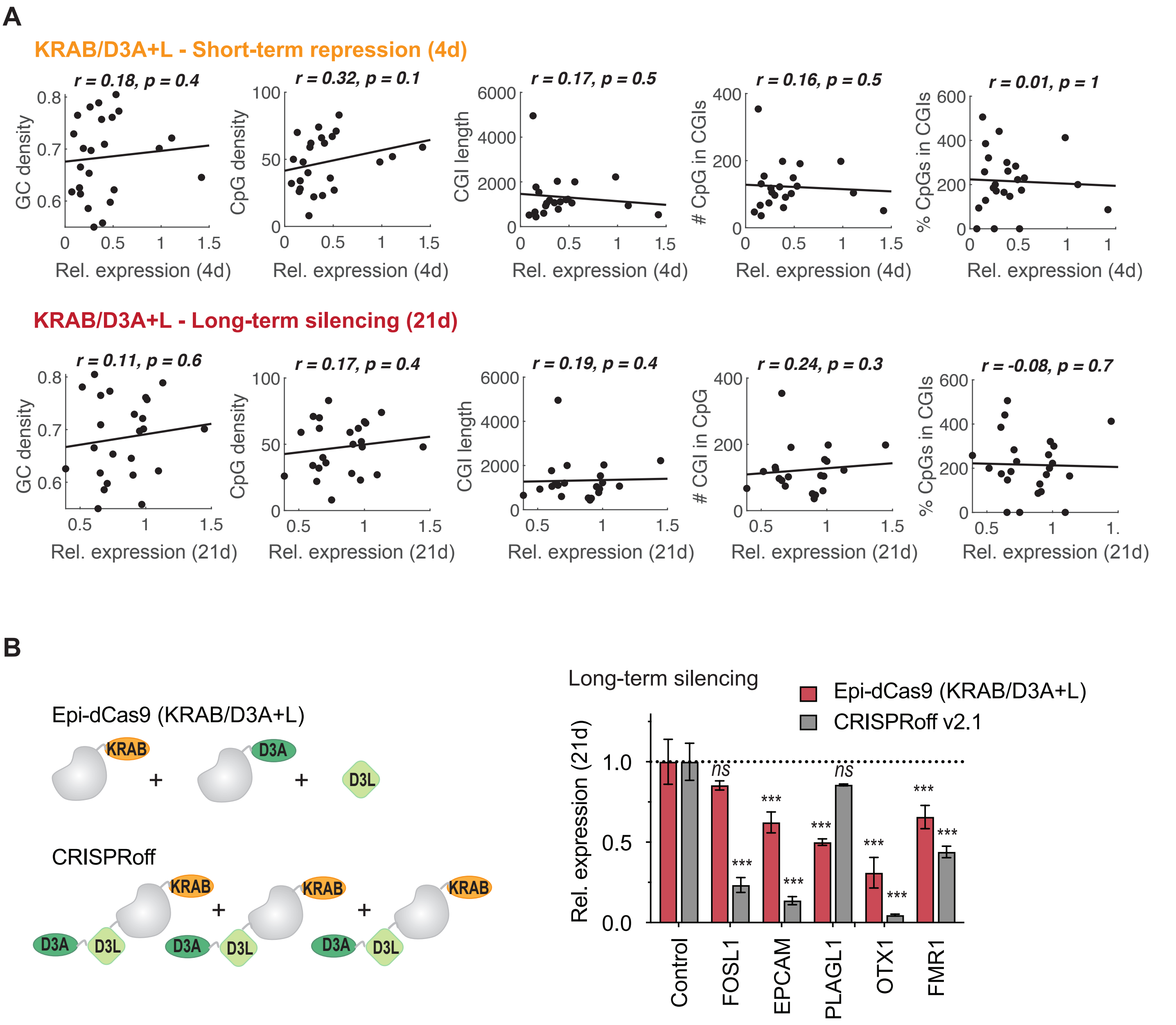

Figure S4. GC context does not predict short or long-term silencing by KRAB/D3A+L epi-dCas9. **A.** Scatterplots of correlation between relative expression and GC density and CpG dinucleotide density of the 250-bp target region as well as parameters of CpG islands (CGIs). Spearman correlation values (*r*) and significance (*p*) are indicated. **B.** Persistent silencing by KRAB/D3A+L epi-dCas9 and CRISPRoff is gene-specific. Cartoon depicting epigenetic editing modulators used in transient transfection assays. KRAB/D3A+L epi-dCas9 is a mixture of equal amounts of each epigenetic editor while CRISPRoff contains three epigenetic editing domains fused to one dCas9 molecule. Target gene silencing was evaluated by RT-qPCR 21 days after co-transfection of plasmids expressing KRAB/D3A+L epi-dCas9 or CRISPRoff v2.1 and 4 targeting gRNAs (n=3, error bar is +/- SEM). Gene expression has been normalized to *GAPDH* and compared to the control without targeting gRNAs (***$p<0.001$, Sidak's multiple comparisons test). Long-term silencing ability of both platforms varies by genomic location.

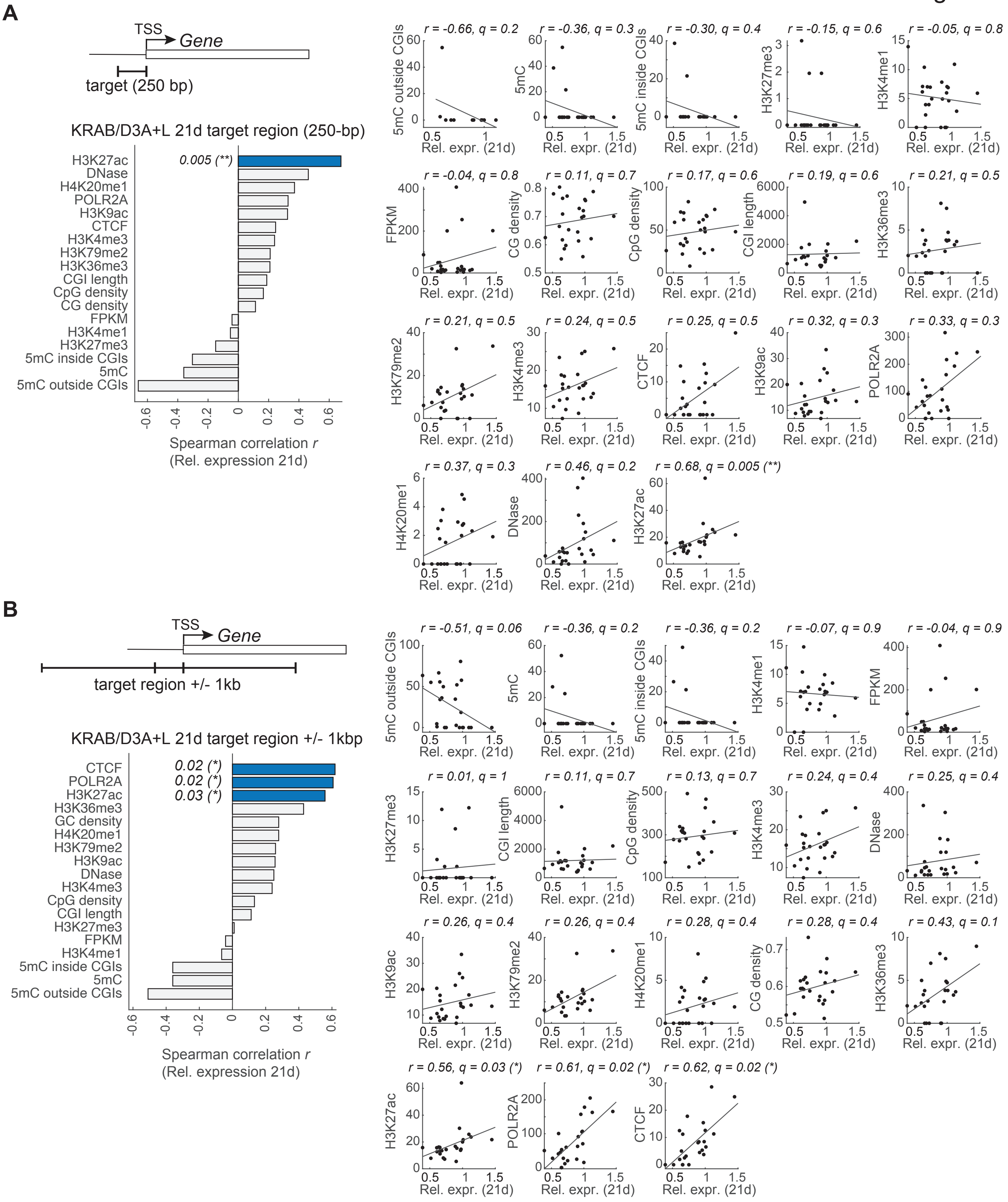

Figure S5. Distinct cis chromatin features correlate with long-term silencing by KRAB/D3A+L epi-dCas9. Bar graph and scatter plots of Spearman correlation (*r*) of different chromatin features with relative expression at long-term time point (21d) at **(A)** the 250-bp target region and **(B)** the extended target region of 250-bp +/- 1kb (*$q<0.05$ and **$q<0.01$; Spearman correlation with Benjamini-Hochberg correction).

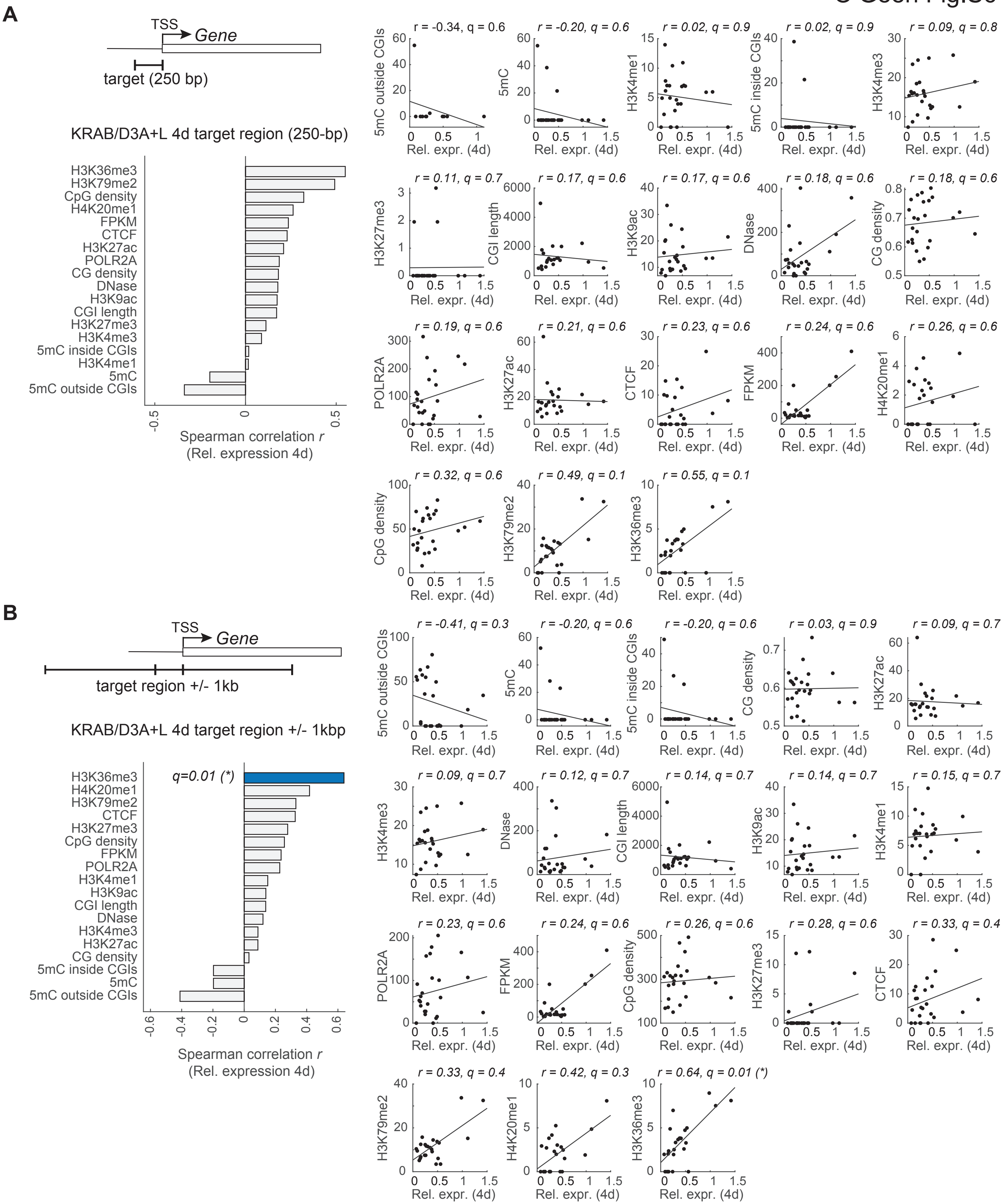

Figure S6. Distinct cis chromatin features correlate with short-term repression by KRAB/D3A+L epi-dCas9. Bar graph and scatter plots of Spearman correlation (r) of different chromatin features with relative expression at short-term time point (4d) at **(A)** the 250-bp target region and **(B)** the extended target region of 250-bp +/- 1kb (**q<0.05*; Spearman correlation with Benjamini-Hochberg correction).

O’Geen Fig.S7

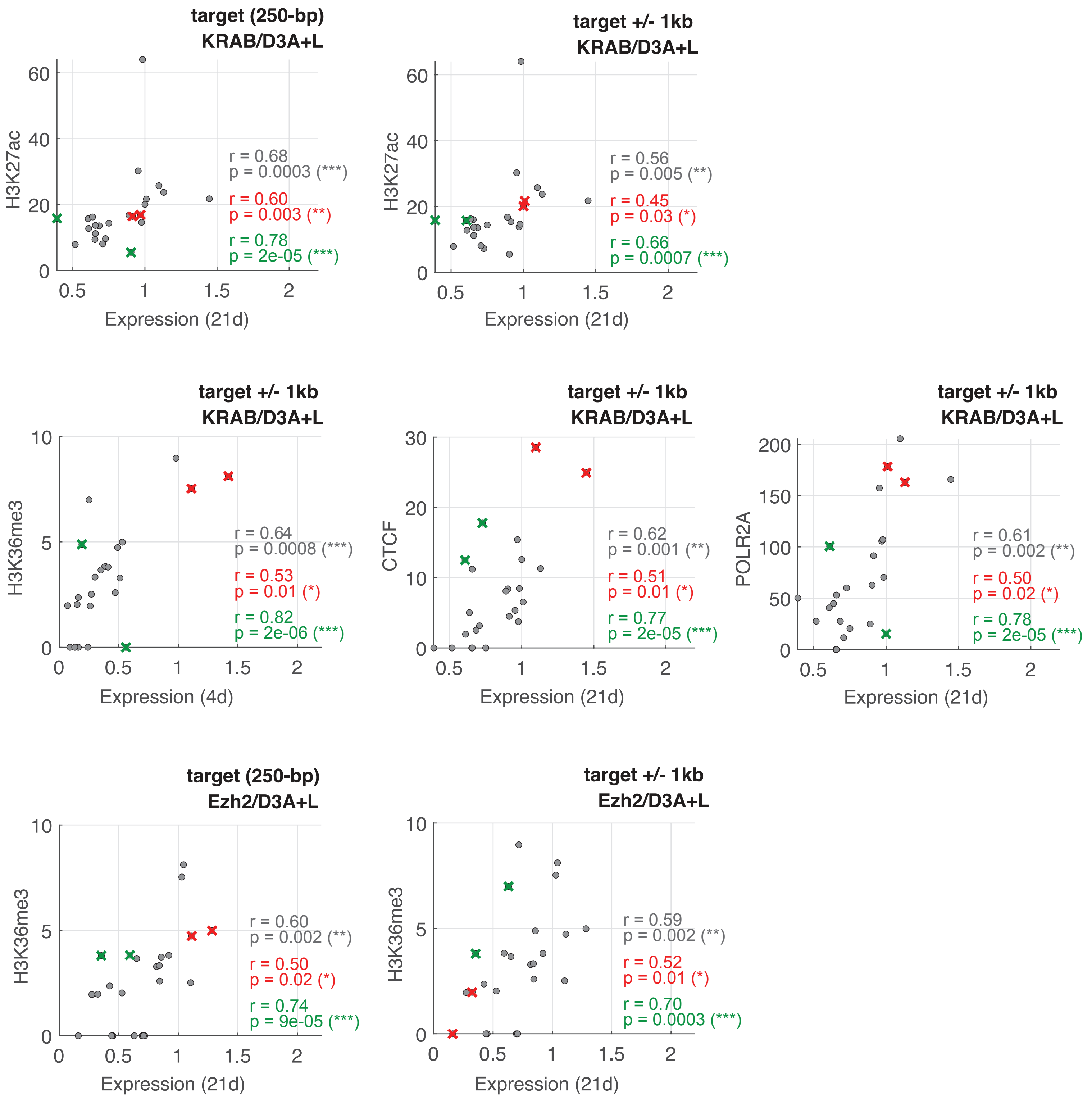

Figure S7. Sensitivity analysis to evaluate robustness of significance of Spearman correlations of relative expression and genomic features. Spearman correlation plots are shown for chromatin features showing significant correlation with short- or long-term expression (q-value<0.05). The results when all data points are included are shown in gray. Values in red denote results after removal of 2 data points, which lead to the highest p-value. Values in green denote results after removal of 2 data points, which lead to the lowest p-value. For instance, in the first plot, we can see that even after removal of any two data points, the Spearman correlation p-value is higher than or equal to 0.003 (r = 0.6).

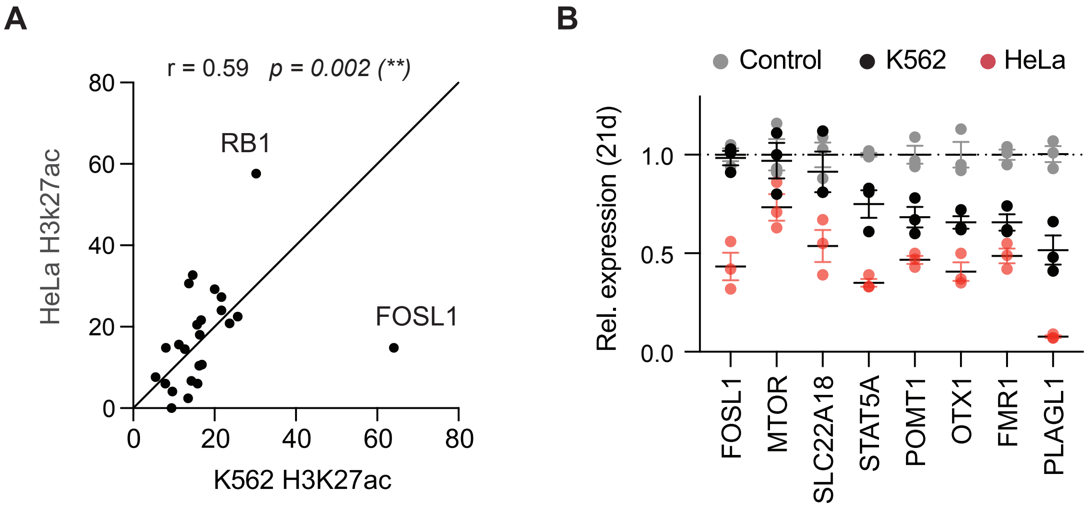

Figure S8. Engineering heritable silencing in HeLa S3 cells. A. Scatter plot of Spearman correlation of H3K27 acetylation enrichment at target gene promoters in Hela S3 and K562 cells. **B.** Target gene silencing in HeLa S3 cells was evaluated by RT-qPCR 21 days after co-transfection of plasmids expressing KRAB/D3A+L epi-dCas9 and 4 targeting gRNAs (n=3, error bar is +/- SEM). Gene expression has been normalized to *GAPDH* and is compared to the control without targeting gRNAs.

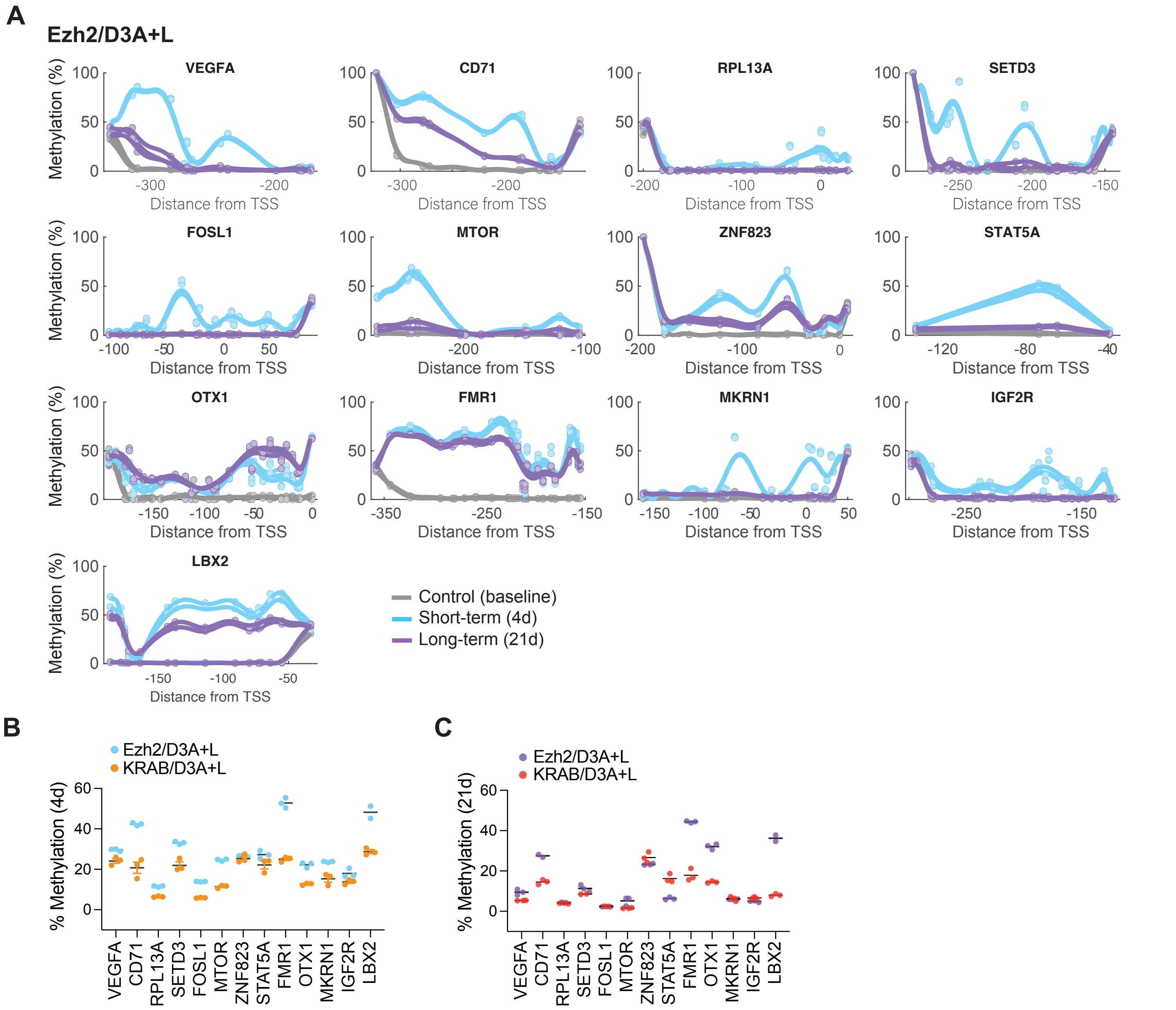

Figure S9. Ezh2/D3A+L epi-dCas9 elicits robust and stable DNA methylation. A. Methylation profiles were determined at baseline, 4 and 21 days after transfection using next-gen bisulfite amplicon sequencing (n=3). Each circle represents a single CpG. Distance relative to TSS is shown on the x-axis. % Mean methylation (n=3) is plotted for 13 genes after KRAB/D3A+L or Ezh2/D3A+L epi-dCas9 treatment at **(B)** short-term (4d) and **(C)** long-term (21d) timepoints.

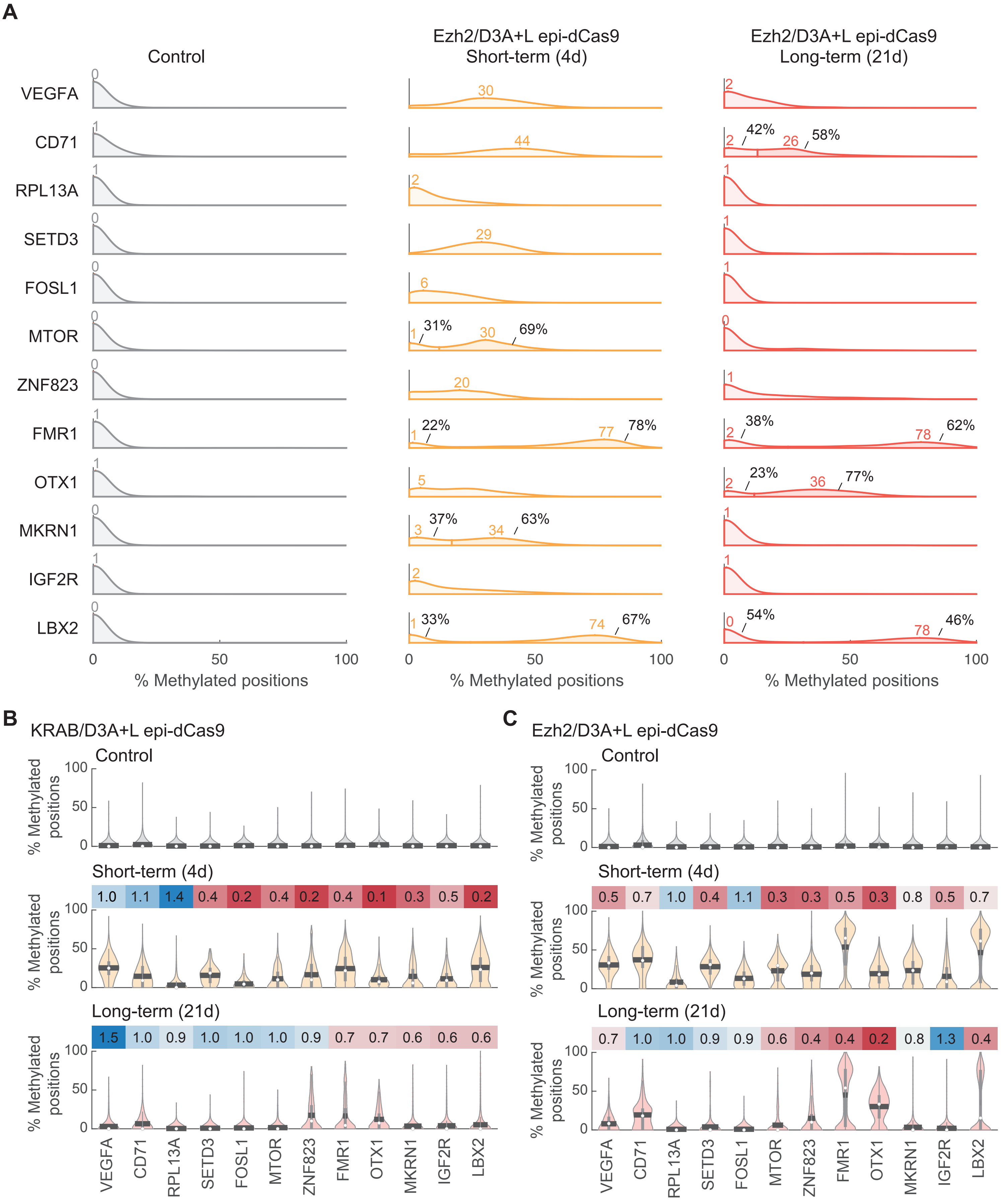

A
Control
Ezh2/D3A+L epi-dCas9 Short-term (4d)
Ezh2/D3A+L epi-dCas9 Long-term (21d)
VEGFA
CD71
RPL13A
SETD3
FOSL1
MTOR
ZNF823
FMR1
OTX1
MKRN1
IGF2R
LBX2
% Methylated positions
B
KRAB/D3A+L epi-dCas9
Control
Short-term (4d)
Long-term (21d)
% Methylated positions
C
Ezh2/D3A+L epi-dCas9
Control
Short-term (4d)
Long-term (21d)
% Methylated positions

Figure S10. Read-level methylation analysis demonstrates enhanced engineering of DNA methylation by Ezh2/D3A+L epi-dCas9. A. Read-level methylation analysis was performed after targeted bisulfite sequencing of 12 promoter regions 4 days and 21 days after Ezh2/D3A+L epi-dCas9 treatment and in untreated control cells. For each read, the percentage of methylated positions was computed and a curve was fitted using ksdensity. Frequency distribution is plotted on the y-axis and is set to the same range for all plots. Distribution is shown for 12 genes for untreated cells as well as for short- and long-term timepoints (4d and 21d, respectively). To determine unimodal and bimodal distributions, peaks and valleys were computed using findpeaks. The numbers denote the mode of the distribution (position of the peak). In this visualization, data from the three replicates are pooled together. Violin plots summarize read-level methylation in untreated control cells, 4 days and 21 days after transient treatment with either **(B)** KRAB/D3A+L or **(C)** Ezh2/D3A+L epi-dCas9. The thick gray line denotes the mean of the distribution, while the median is shown as a white circle and the boxplot represents the 24th -75th percentile of the data. Heatmap illustrates relative short- (4d) and long-term (21d) expression values. Dark red represents high silencing while dark blue represents high resistance to silencing or re-activation.

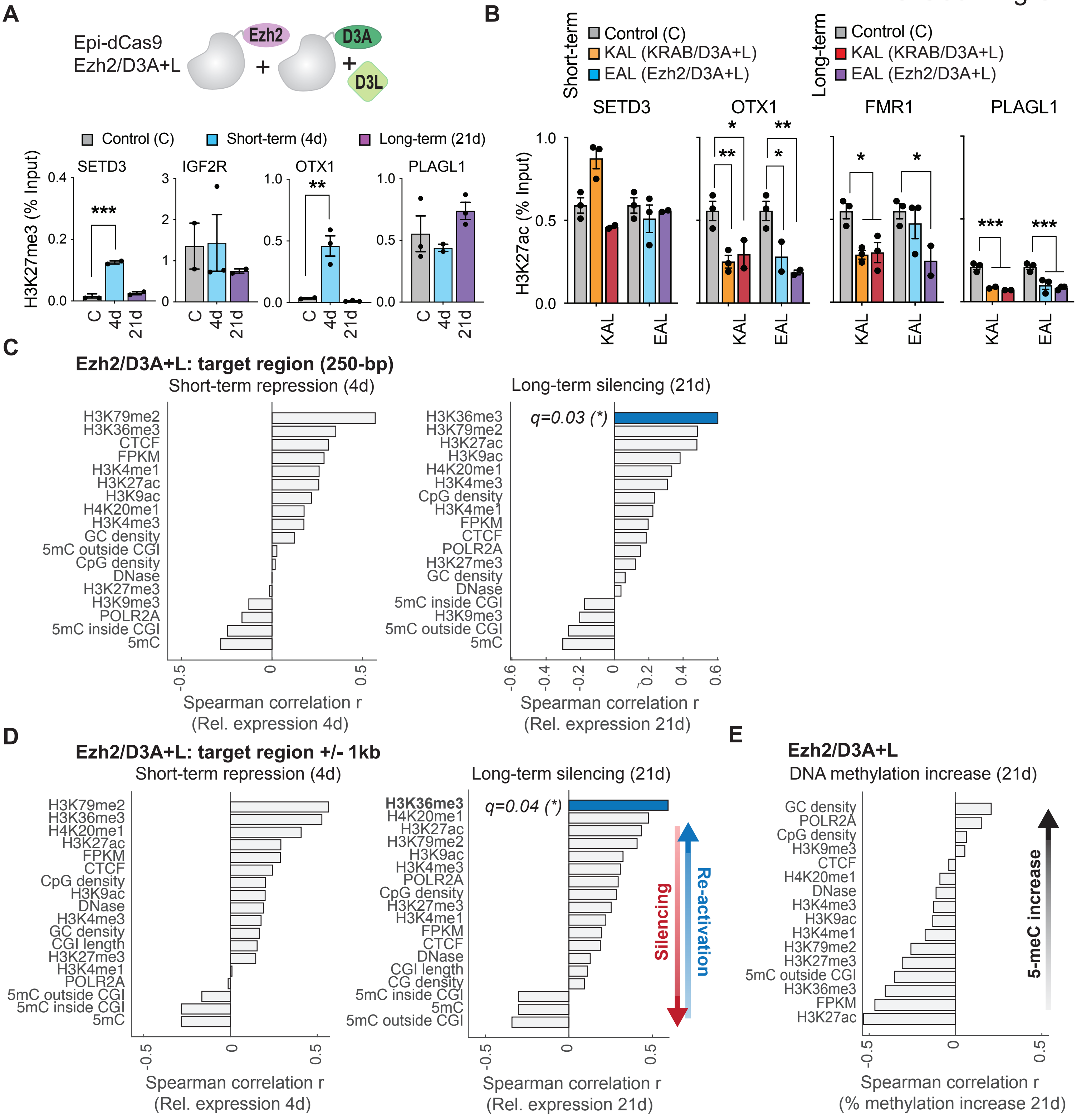

Figure S11. Ezh2/D3A+L epi-dCas9 overcomes dependency on H3K27ac observed with KRAB/D3A+3L. H3K27me3 **(A)** and H3K27ac **(B)** ChIP-qPCR assays were performed 4 and 21 days after transfection with plasmids expressing indicated epi-dCas9 combinations and 4 targeting gRNAs. H3K27me3 ChIP enrichment was calculated relative to input (dCq) and H3K27ac was further normalized to the GAPDH promoter (ddCq). ChIP enrichment was compared to control cells transfected with dCas9 without epigenetic editing domains (n=3, error bar is +/- SEM, *$p<0.5$, **$p<0.01$, ***$p<0.001$). Correlation of chromatin features was determined for short-term repression and long-term silencing in the **(C)** 250-bp target region and **(D)** extended target region (250-bp target region +/- 1kb). Spearman correlation values were adjusted by Benjamini-Hochberg correction. Significant adjusted p-values (*$q < 0.05$) are indicated with asterisk. Positive Spearman correlation r indicates correlation with re-activation of target genes. **E.** Correlation of chromatin features with heritable methylation increase (21d) in the 250-bp target region.

O'Geen Fig.S12

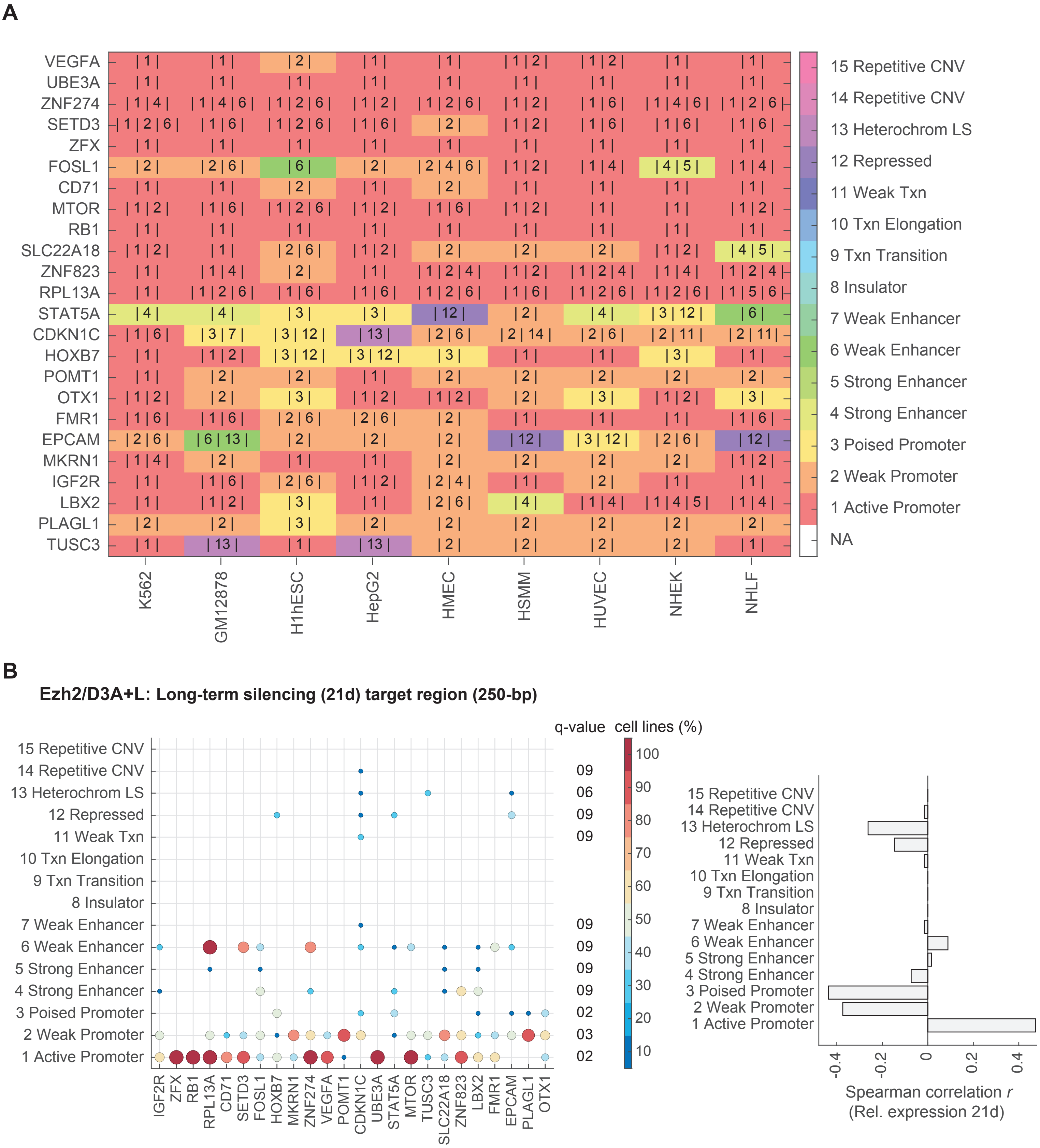

Figure S12. Persistent silencing by Ezh2/D3A+L epi-dCas9 is indifferent to divergent chromatin state. **A.** Matrix listing 15 chromHMM states across 9 cell lines (columns) and 24 regions (rows). All chromHMM states overlapping with the target region in the given cell line are listed in the matrix. The color represents the state with the lowest index (e.g., all cell lines with state “1 active promoter” being present in the target region are shown in red). **B.** For each of the 15 chromHMM states (rows) and 24 regions (columns), the color and size of the circle represents frequency at which the chromatin state is observed across 9 cell types in the target region. For instance, *MTOR* target region contains state 1 in 9/9=100% cell lines, state 2 in 5/9=56% cell lines, and state 6 in 3/9=33% cell lines. The columns are sorted by the relative expression at 21d for Ezh2/D3A+L. The q-value column shows statistical significance of Spearman correlation of this frequency and the relative expression at 21d for Ezh2/D3A+L, adjusted using Benjamini-Hochberg correction. Bar graph on the right shows r values of the Spearman correlation.
